# A cell death program–based tumor signature stratifies prognosis, immune landscape, and therapeutic response in glioma

**DOI:** 10.3389/fonc.2026.1824504

**Published:** 2026-05-21

**Authors:** Huaichao Zhang, Zijiang Yang, Qiang Xie, Pin Chen, Jinlong Huang, Shuang Liu, Zeyang Li, Wei Sun, Xiaobiao Zhang, Tao Xie

**Affiliations:** Department of Neurosurgery, Zhongshan Hospital, Fudan University, Shanghai, China

**Keywords:** Apoptosis, glioma, microenvironment, prognosis, pyroptosis

## Abstract

**Background:**

Glioma exhibits profound intratumoral heterogeneity driven by coordinated cell death programs and dynamic tumor–microenvironment interactions. However, the transcriptional landscape linking inflammatory cell death signatures with malignant cell states, genomic alterations, and therapeutic vulnerability remains incompletely defined. We sought to develop a prognostic cell death–associated signature and elucidate its biological and clinical relevance in glioma.

**Methods:**

Using transcriptomic and clinical data from TCGA-GBMLGG and external validation cohorts, we constructed a pyroptosis–apoptosis–associated gene signature (PA.Sig) through integrative survival modeling. Genomic alteration profiling, pathway enrichment analyses, and tumor immune microenvironment characterization were performed. Single-cell RNA sequencing datasets were analyzed to delineate malignant cell states and intercellular communication patterns. Functional validation was conducted in glioma cell lines treated with chemotherapeutic agents.

**Results:**

PA.Sig stratified patients into high- and low-risk groups with significantly different overall survival and independent prognostic value. High-risk tumors were characterized by increased somatic mutation burden, enrichment of cell cycle and inflammatory pathways, and a malignant-centered outgoing signaling architecture. Single-cell analyses revealed preferential enrichment of MES-like and AC/MES-like states in high-risk tumors, accompanied by intensified ligand–receptor interactions with immune and stromal compartments. Despite increased immune infiltration, high-risk tumors displayed features of immune dysfunction and exhaustion. *In vitro*, chemotherapy induced concomitant apoptotic and pyroptotic morphologies in glioma cells, supporting coexistence of lytic and non-lytic death programs. Collectively, PA.Sig captured a coordinated inflammatory cell death landscape associated with aggressive cellular states and immune remodeling.

**Conclusions:**

Our study defines a clinically relevant inflammatory cell death signature that integrates tumor cell state plasticity, genomic instability, and immune microenvironment dynamics in glioma. PA.Sig may serve as a prognostic biomarker and provide a rationale for combinatorial strategies targeting cell death pathways and immune modulation.

## Introduction

Gliomas are the most common primary malignant tumors of the central nervous system and remain associated with dismal clinical outcomes despite advances in surgical techniques, radiotherapy, chemotherapy, and molecular classification systems (1, 2). The incorporation of isocitrate dehydrogenase (IDH) mutation and 1p/19q codeletion into the World Health Organization classification has substantially refined diagnostic and prognostic stratification (3, 4). However, even within molecularly defined categories, patients exhibit marked heterogeneity in therapeutic response, patterns of recurrence, and overall survival (5, 6). These observations underscore the need for biologically grounded frameworks that extend beyond static genomic alterations to capture dynamic tumor states relevant to treatment response and disease evolution.

Regulated cell death is a fundamental biological process governing tissue homeostasis, immune surveillance, and stress adaptation (7, 8). In cancer, dysregulation of cell death programs enables tumor cell survival under genotoxic stress and therapeutic pressure (9). While apoptosis has long been recognized as a central anticancer mechanism, inflammatory forms of regulated cell death—particularly pyroptosis—have emerged as critical modulators of tumor–immune interactions (10–12). Pyroptosis is characterized by gasdermin-mediated membrane pore formation and the release of proinflammatory cytokines and damage-associated molecular patterns (DAMPs), thereby reshaping the tumor microenvironment (13, 14). In glioma, individual components of apoptotic and pyroptotic pathways have been associated with tumor progression, immune infiltration, and treatment resistance (15–17). However, most studies have evaluated these pathways in isolation, without considering the possibility that coordinated activation of multiple cell death programs may define integrated tumor states with distinct biological and clinical implications.

Emerging evidence indicates that tumors can adopt stable or semi-stable cellular states characterized by coordinated transcriptional programs rather than single-gene alterations (18, 19). Such states may arise in response to intrinsic oncogenic stress, immune-mediated selection, or therapeutic intervention, and can shape genomic evolution, immune composition, and drug sensitivity (20, 21). In glioma, profound intratumoral heterogeneity and adaptive plasticity are well documented at both the bulk and single-cell levels (22, 23). Yet it remains unclear whether coordinated cell death programs delineate reproducible tumor states across patients and disease stages, and whether such states are linked to specific genomic backgrounds, immune remodeling patterns, and histopathological features.

The tumor immune microenvironment plays a central role in glioma biology and therapeutic response (24, 25). Although immune checkpoint blockade has demonstrated limited efficacy in unselected glioma populations, subsets of patients exhibit signs of immune activation and clinical benefit (26, 27). Reliable biomarkers capable of identifying immunologically active or therapy-sensitive tumors are still lacking. Given the immunogenic nature of inflammatory cell death, coordinated cell death programs may influence immune cell recruitment, activation status, and functional polarization within the glioma microenvironment (28, 29). Furthermore, therapeutic agents such as temozolomide can induce stress responses that intersect with cell death signaling, potentially driving state transitions during disease progression and recurrence (30, 31). Thus, cell death–associated tumor states may represent an integrative node linking intrinsic tumor biology, immune contexture, and treatment adaptation.

In this study, we hypothesized that coordinated cell death programs define a biologically meaningful tumor state in glioma that shapes immune remodeling, genomic alterations, histopathological characteristics, and therapeutic response. To test this hypothesis, we systematically characterized cell death program patterns by integrating bulk transcriptomic datasets, single-cell RNA sequencing, somatic mutation profiles, and whole-slide histopathological imaging. We identified a reproducible cell death program–defined tumor state associated with distinct immune signaling architecture, mutation landscapes, and clinical outcomes. Our findings provide a multidimensional framework linking regulated cell death to tumor state biology and suggest potential applications for patient stratification and precision oncology in glioma.

## Method

### Construction of the PA signature

First, 113 PA-related genes were collected from published literature. Univariate Cox analysis was performed based on the 113 PA-related genes to identify the overall survival (OS)-related genes in TCGA-GBMLGG. Then, least absolute shrinkage and selection operator (LASSO) regression was conducted to further screen genes. Subsequently, Friends and random survival forest (RSF) algorithms were employed to identify the top 10 genes of importance, respectively. Last, an intersection was conducted to obtain the final genes, called PA.Sig.

### Bulk transcriptomic data preprocessing

Bulk RNA-sequencing data and corresponding clinical information for lower-grade glioma (LGG) and glioblastoma multiforme (GBM) patients were obtained from the public UCSC Xena data portal (https://xenabrowser.net/). Specifically, we downloaded the gene expression STAR-Count datasets and phenotype data from the “GDC TCGA Lower Grade Glioma (LGG)” and “GDC TCGA Glioblastoma (GBM)” cohorts. Raw counts were converted to transcripts per million (TPM) values using gene length information, followed by log2 transformation (log2[TPM + 1]). Ensembl gene identifiers were mapped to HGNC gene symbols using the biomaRt package, and duplicated symbols were aggregated by summation. Samples lacking complete survival or clinical information were excluded from downstream analyses. Batch effects between datasets were assessed and addressed where appropriate. The molecular annotation data for MGMT promoter methylation status and IDH mutation status were obtained from the same UCSC Xena resource, Illumina Human Methylation 450 and Ensemble Somatic Variant (WXS) corresponding. For independent external validation, bulk RNA-sequencing data (mRNAseq_693, read counts) and comprehensive clinical annotation for glioma patients were obtained from the Chinese Glioma Genome Atlas (CGGA) portal (http://www.cgga.org.cn).

### Construction of the cell death program–based tumor state

A curated gene set encompassing pyroptosis-related genes and intrinsic and extrinsic apoptosis pathways (113 PA-related genes) was compiled based on prior literature. In the TCGA training cohort, univariate Cox proportional hazards regression was first applied to identify genes significantly associated with overall survival. Genes passing the predefined significance threshold were subjected to least absolute shrinkage and selection operator (LASSO) Cox regression using 10-fold cross-validation to prevent overfitting. The 24 genes were retained based on the optimal penalty parameter (λ) determined in the LASSO-Cox regression model using 10-fold cross-validation. Specifically, we selected the λ value corresponding to the minimum mean cross-validated partial likelihood deviance (lambda.min), which provides the best model fit while minimizing overfitting. At this λ value, 24 genes with non-zero coefficients were retained and used for subsequent model construction.

A cell death program score was calculated for each sample as a linear combination of normalized gene expression values weighted by their corresponding LASSO coefficients. Scores were Z-score standardized, and an optimal cutoff value was determined using maximally selected rank statistics to stratify patients into high and low cell death program states. The prognostic performance of this stratification was validated in the independent CGGA cohort using identical coefficients and cutoff strategy.

### Clinical annotation and molecular feature integration

Clinical variables, including age, sex, tumor grade, treatment information, and survival outcomes, were harmonized across datasets. IDH mutation status was inferred from somatic mutation data by identifying canonical hotspot mutations in IDH1 and IDH2. MGMT promoter methylation status was estimated using beta values from Illumina 450K methylation arrays based on established promoter-associated probes.

Somatic mutation profiles were analyzed using the maftools framework. Tumor mutational burden (TMB) was calculated as the number of nonsynonymous mutations per megabase. Mutational landscapes, co-occurrence, and mutual exclusivity patterns were compared between cell death program–defined tumor states. Survival analyses incorporating combined molecular and state-based stratifications were performed using Kaplan–Meier estimates and log-rank tests.

### Immune microenvironment analysis

The tumor immune microenvironment was characterized using multiple orthogonal computational deconvolution methods, including TIMER, CIBERSORT-ABS, EPIC, MCP-counter, quanTIseq, and xCell. Immune and stromal scores, as well as tumor purity estimates, were obtained using the ESTIMATE algorithm. Differences in immune cell infiltration and microenvironmental features between tumor states were assessed using nonparametric statistical tests. Concordance across deconvolution methods was emphasized to ensure robustness of immune associations.

### Single-cell data preprocessing

Cell type annotated scRNA data for this manuscript was download from the single-cell portal with study ID SCP2389 at https://singlecell.broadinstitute.org/single_cell/study/SCP2389/programs-origins-and-niches-of-immunomodulatory-myeloid-cells-in-human-gliomas. Malignant cells and T cells were extracted individually. Following standard preprocessing, normalization, and clustering pipelines, cell state identification was performed based on the expression of well-established marker gene signatures. For malignant cells, five core gene signatures were compiled from the literature to represent key cellular populations:

Oligodendrocyte Progenitor Cell (OPC): PDGFRA, CSPG4, OLIG1, OLIG2, SOX10, NKX2-2, DLL3, BCAN, GPR17.
Neural Progenitor Cell (NPC): SOX4, SOX11, DCX, ASCL1, NEUROD1, NEUROD2, HES6, DLL3, TUBB3.
Astrocyte-like (AC): GFAP, AQP4, ALDH1L1, SLC1A3, CLU, APOE, FABP7, GLUL, SOX9.
Mesenchymal-like (MES): CHI3L1, CD44, ANXA1, LGALS3, VCAN, S100A10, EMP1, PLAU, ITGA5, FN1.
Cycling (CYC): MKI67, TOP2A, CENPF, TYMS, HMGB2, UBE2C, CCNB1, CCNB2, CDC20.

For T cells, eight core gene signatures were compiled:

Naïve T cells (T_naive): CCR7, IL7R, LTB, TCF7, LEF1, MAL.
Cytotoxic T cells (T_cytotoxic): NKG7, CTSW, PRF1, GZMB, GZMH, IFNG.
Exhausted T cells (T_exhaust): PDCD1, LAG3, HAVCR2, TIGIT, CTLA4, TOX, EOMES.
Regulatory T cells (T_reg): FOXP3, IL2RA, CTLA4, IKZF2, TIGIT.
γδ T cells (T_gd): TRDC, TRGC1, TRGC2.
Cycling T cells (T_cycling): MKI67, TOP2A, CENPF, HMGB2, TYMS.
Interferon-responsive T cells (T_ifn): ISG15, IFIT1, IFIT3, STAT1, CXCL10.
Tissue-resident memory T cells (T_trm): ITGAE, CXCR6, ZNF683, RUNX3.

Module scores for each signature were calculated for every individual cell using the AddModuleScore function in Seurat. To annotate the transcriptional clusters identified by Seurat clusters, the average module score for each of the five signatures was computed per cluster. Each cluster was then assigned a dominant cellular state based on its highest average signature score.

### Single-cell trajectory inference

To characterize dynamic cellular state transitions associated with the cell death program–defined tumor state, pseudotemporal trajectory analysis was performed on malignant tumor cells using Monocle3. Following cell-type annotation, malignant cells were subsetted and converted from Seurat objects to Monocle3 cell data sets using raw count matrices and corresponding metadata. Dimensionality reduction was conducted using principal component analysis, followed by batch alignment where appropriate. Cell trajectories were inferred using the learned principal graph, and pseudotime ordering was calculated to reflect continuous transcriptional state transitions.

The cell death program score was projected onto single cells by calculating weighted expression scores using the same gene coefficients derived from the bulk model. Changes in cell death program activity along pseudotime were examined to assess whether this tumor state represents a dynamic continuum rather than a static classification. Differential gene expression analysis along pseudotime was performed to identify biological processes associated with state transitions.

### Cell–cell communication analysis

To investigate how the cell death program–defined tumor state influences intercellular signaling within the tumor microenvironment, cell–cell communication analysis was conducted using the CellChat framework. Annotated single-cell datasets were used to infer ligand–receptor interactions among malignant cells and major immune and stromal cell populations. Communication probability was computed based on the expression of known ligand–receptor pairs, and signaling networks were constructed for samples stratified by cell death program state.

Differences in overall communication strength, signaling pathway activity, and cell-type–specific interaction patterns were compared between tumor states. Key signaling pathways associated with immune modulation and tumor–immune interactions were further examined to identify state-specific communication programs potentially linked to immune remodeling and therapeutic response.

### Western blot

After mixed with phosphatase and protease inhibitors (Thermo Fisher Scientific, Waltham, Massachusetts, USA), RIPA buffer was used to lyse resected tumors or normal brain tissues.

Protein concentration was quantified by the BCATM Protein Assay Kit (Fremont Thermal Science, California, USA). After calculation, equal proteins were isolated using sodium dodecyl sulfate-polyacrylamide gel electrophoresis (SDS-PAGE). Then each bands was transferred onto a polyvinylidene fluoride (PVDF) membrane. We incubated the membrane with the primary antibody, blocked with 5% milk at 37°C for 60 min, overnight. After washed by PBS, the membrane was incubated with the secondary antibody. Finally, we used the ECL system (Perkin Elmer, Waltham, MA, USA) scan the membranes and the pictures were processed using software ImageJ. Antibodies used were listed (anti-GSDME, #GSDME antibody Abcam EPR 19859, Abcam, Cambridge, UK; anti-cleaved N-terminal GSDME, #GSDME-N antibody Abcam EPR 20866-160, Abcam, Cambridge, UK; anti-GSDME-C-terminal, #GSDME-C antibody Abcam EPR 19859-60, Abcam, Cambridge, UK; anti-Caspase-3, #14220, Cell Signaling Technology, Boston, USA; anti-cleaved-Caspase-3 (Asp175), #9664, Cell Signaling Technology, Boston, USA; anti-cleaved-Caspase-9 (Asp330), #52873, Cell Signaling Technology, Boston, USA; anti-cleaved-Caspase-8 (Asp387), #8592, Cell Signaling Technology, Boston, USA; anti-cleaved-Caspase-7 (Asp198), #9491, Cell Signaling Technology, Boston, USA; anti-beta Actin, #beta Actin antibody mAbcam 8226, Abcam, Cambridge, UK).

### Cell viability and cytotoxicity

The cell viability was detected by Cell Titer-Glo^®^ Luminescent Cell Viability Assay (Promega) to sense the quantity of intracellular ATP amount. Cytotoxicity was detected by CytoTox 96^®^ Non-Radioactive Cytotoxicity Assay (Promega) to measure the LDH release. All steps were according to the protocol provided by the supplier.

### Electron microscopy

The morphology of mitochondria was imaged through transmission electron microscope using Hitachi HT7800 (Hitachi, Japan). Glioma cells were fixed with 2.5% glutaraldehyde and 1% osmium tetroxide at 4 °C and immersed in spur resin after dehydration. Ultrathin sections (60-80nm) were made by a Leica UC7 ultramicrotome (Leica, Germany). The cells were then stained with 4% uranyl acetate and lead citrate. Finally, images were captured using a Hitachi HT7800 (Hitachi, Japan) transmission electron microscope.

2.5% glutaradehyde was used to fix glioma cells on the overslips overnight at 4 °C. Then, a graded series of alcohol (30, 50, 70, 80, 95, and 100% ethanol) was used to dehydrate the overlips. After dehydration in alcohol, the overlips were dried with a critical point drier. The dried samples were viewed under a ZEISS GeminiSEM300 (ZEISS, Germany) scanning electron microscope at 5000 magnifications after sputter-coating with gold.

### Apoptosis detected by flow cytometry

Following treatment of chemotherapy drugs, the surviving cells were stained with Propidium Iodide (PI) dyes and FITC-Annexin V (Servicebio, Shanghai, China) under dark at 37 °C for 30min. Then the cells were analyzed using an EXFLOW-206 cytometer (Dakewe, Shenzhen, China).

### TUNEL assay

U251 cells after transfections were seeded in 6-well plates at 1000 cells per well and treated with 200uM TMZ or PBS for 48h. Briefly, after digested with proteinase solution (Servicebio, Shanghai, China) under room temperature for 20min, sections were immersed in TdT labelling buffer, dUTP and TdT enzyme with a ratio of 50:5:1. Sections were treated with DAPI for nucleus counterstain. Fluorescence was evaluated at 400 × magnification under a fluorescent microscope (Olympus, Tokyo, Japan).

### Assessments of intracellular ROS

U251 cells were digested with proteinase solution and centrifuged. Then, the cells were resuspended in Dichlorofluorescin-diacetate (DCFH-DA) solution (10umol/L) to reach a final concentration of 1-20×106/L and incubated for 30min in the dark at 37 °C. After wash out the extracellular probes, the DCF were detected under the FITC parameters. Images were acquired via a high-throughput confocal microscope (Olympus, Tokyo, Japan).

### Mitochondrial membrane potential (ψ_mito_) measurements

The mitochondrial membrane potential of U251 cells was detected using the mitochondrial membrane potential detection kit (JC-1; Solarbio, Beijing, China) and Tetramethylrhodamine Methyl Ester assay kit (TMRM; abcam, America). Briefly, U251 cell suspensions (1×10^6^/ml) were incubated with JC-1 dye at room temperature for 30 min. Then the cells were washed twice with dye buffer and detected by flow cytometry (Dakewe, Shenzhen, China). The green JC-1 monomers (Ex/Em 490/530, indicative of loss) and orange JC-1 aggregates (Ex/Em 525/590, indicative of normal) was detected using carbonyl cyanide 3-chlorophenylhydrazone (CCCP) as positive control and cells without JC−1 staining as negative control.

For TMRM assay, U251 cells was seeded into six-well plates and harvest after drugs treatment. Following incubated with 100nM TMRM or 20 uM FCCP (a positive control) for 30 min at room temperature, adherent cells was observed via a confocal microscope (Olympus, Tokyo, Japan).

### Statistical analysis

All statistical analyses were conducted using R. Survival differences were evaluated using Kaplan–Meier analysis and Cox proportional hazards models. Continuous variables were compared using Wilcoxon rank-sum tests, and categorical variables were assessed using chi-square or Fisher’s exact tests, as appropriate. Multiple testing correction was applied where necessary. Statistical significance was defined as a two-sided P value < 0.05 unless otherwise specified.

## Results

### Identification of the mode of glioma cell death *in vitro*

**Figure 1:**
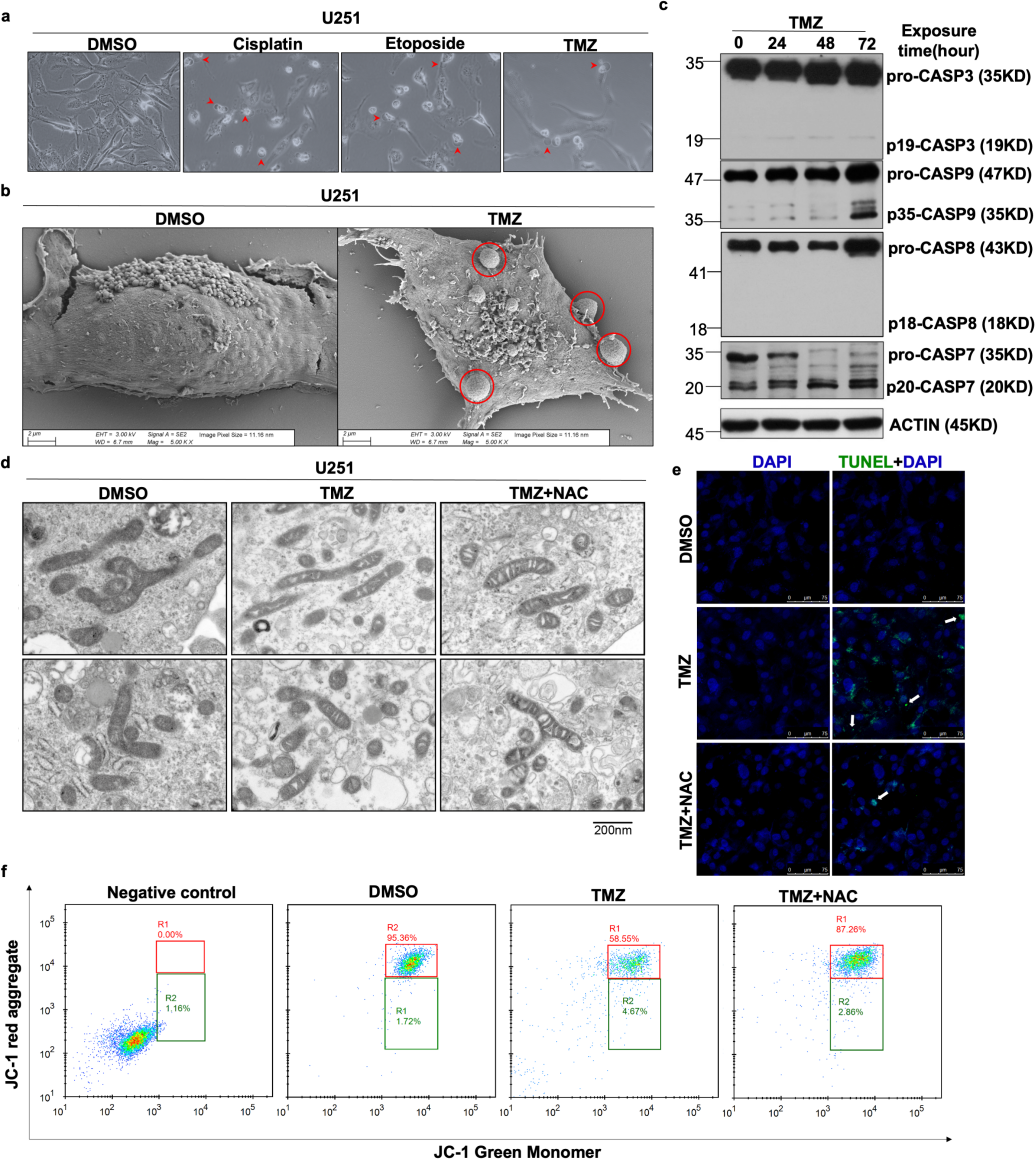
Identification of the mode of glioma cell death *in vitro*. **(A)** Morphologic change of U251 cultured with DMSO, cisplatin, etoposide and TMZ after 48h. **(B)** Scanning electron microscope of tumor cells treated with DMSO (left) and TMZ (right). **(C)** Immunoblotting demonstrates caspase-3, caspase-9 and caspase-7 was cleaved, while caspase-8 was not, in U251 cells after TMZ treated. **(D)** Representative transmission electron microscopy (TEM) images of mitochondria in U251 cells treated with DMSO, TMZ or TMZ+NAC for 48 h. **(E)** Tunnel staining after DMSO, TMZ and TMZ +NAC treatment was detected by immunofluorescence. **(F)** Flow cytometry analysis showing TMZ affect the aggregation of JC-1 in U251 cells, which could be prevent by simultaneously treated with NAC.

To characterize the mode of cell death induced by chemotherapeutic agents, U251 glioma cells were treated with cisplatin, etoposide, and temozolomide (TMZ), followed by morphological assessment (**Figure 1A**). Treated cells exhibited concurrent morphological features characteristic of both pyroptosis and apoptosis. Scanning electron microscopy (SEM) provided high-resolution evidence of pyroptotic body formation, a defining ultrastructural hallmark of pyroptosis (**Figure 1B**). Western blot analysis showed increased levels of cleaved Caspase-3, Caspase-7, and Caspase-9 following TMZ treatment, indicating activation of caspase-related apoptotic signaling (**Figure 1C**). Transmission electron microscopy further revealed pronounced mitochondrial deformation in TMZ-treated U251 cells, which was partially reversed by N-acetylcysteine (NAC), indicating redox-dependent mitochondrial injury (**Figure 1D**). To determine whether mitochondrial alterations were associated with reactive oxygen species (ROS) dysregulation, intracellular ROS levels were quantified using DCFH-DA staining and flow cytometry. TMZ treatment (200 μM, 48 h) significantly increased ROS levels compared with controls (mean fluorescence intensity: 1.32 ± 0.05 vs 1.02 ± 0.10, P < 0.05), whereas NAC co-treatment markedly attenuated ROS accumulation (**Supplementary Figure 1A**). TUNEL staining demonstrated substantial DNA damage following TMZ exposure, which was mitigated by NAC treatment (**Figure 1E**), supporting ROS-mediated genotoxic stress. Given that excessive ROS disrupts mitochondrial function, mitochondrial membrane potential (MMP) was assessed using JC-1 staining. TMZ significantly reduced mitochondrial JC-1 aggregation, indicating MMP loss, whereas NAC restored mitochondrial polarization (**Figure 1F**). Consistently, TMRM staining revealed a marked decrease in mitochondrial fluorescence intensity following TMZ treatment compared with DMSO controls (1.44 ± 0.06 vs 14.68 ± 0.22, P < 0.001). NAC co-treatment partially rescued mitochondrial fluorescence (3.77 ± 0.05 vs 1.44 ± 0.06, P < 0.01). As a positive control, FCCP completely abolished mitochondrial membrane potential, confirming assay specificity (**Supplementary Figure 1B**).

Collectively, these findings indicate that TMZ induces concurrent apoptotic and pyroptotic cell death in glioma cells, accompanied by ROS accumulation and mitochondrial dysfunction.

### Construction of molecular subtypes based on the PA-related genes

**Figure 2:**
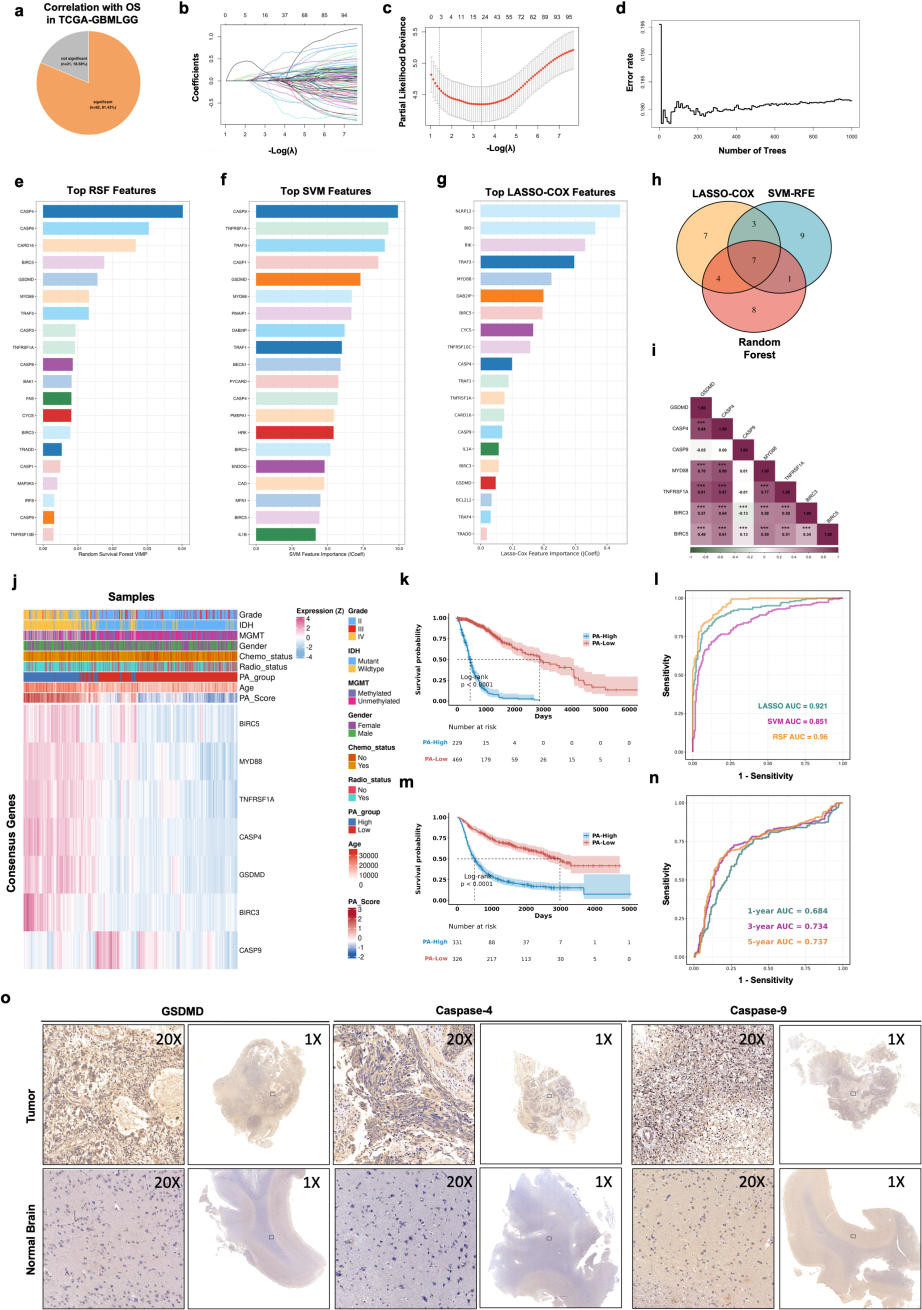
Construction of molecular subtypes based on the PA-related genes. **(A)** The pie chart showing the number and proportion of PA-related genes significantly associated with OS performed by univariate Cox regression in TCGA-GBMLGG. **(B, C)** Lasso further screening the prognostic genes. **(D, E)** RSF identifying the top 20 most important genes based on the result of lasso. **(F)** SVM analysis identifying the top 20 most important genes based on the result of lasso. **(G)** Lasso-Cox analysis identifying the top 20 most important genes. **(H)** The intersection of the results of Lasso-Cox, RSF and SVM analysis. **(I)** Spearman correlation between genes in PA.Sig. **(J)** Heatmap showing the association between the expression of genes in PA.Sig and risk score, as well as some clinical indicators in TCGA-GBMLGG. K-M curves of OS stratified by risk score in **(K)** TCGA-GBMLGG and **(M)** CGGA_693. Log-rank test was used to generate the p values. **(L)** Comparison of the ROC curves of the 3-year survival expectations of three different models. **(N)** Validation of the model in CGGA_693 by comparison of 1-year, 3-year, and 5-year ROC curves. **(O)** Representative IHC staining images of GSDMD and Caspase-4 in glioma and normal brain tissues. (***p<0.001).

To systematically investigate the prognostic relevance of pyroptosis- and apoptosis-related pathway in glioma, a comprehensive set of candidate genes (113 PA-related genes) was first curated. Functional enrichment showed that these genes were involved in programed cell death and inflammatory signal, such apoptosis, NF-κB signal and inflammasome (**Supplementary Figure 2**). Univariate Cox regression analysis in the TCGA-GBMLGG cohort revealed that the majority of these genes were significantly associated with overall survival, with 92 genes (92/113, 81.42%) showing prognostic significance (**Figure 2A**). This widespread survival association suggests that dysregulation of cell death–related pathways is broadly involved in glioma progression and provides a strong rationale for integrative multigene modeling. Subsequently, LASSO-Cox regression was used to further screen the genes, and 24 genes were retained (**Figures 2B, C**). In parallel, random survival forest (RSF) (**Figures 2D, E**) and survival-support vector machine (SVM) (**Figure 2F**) models were constructed to evaluate feature importance from complementary analytical perspectives. A subset of consensus genes, including GSDMD, CASP4, CASP9, MYD88, TNFRSF1A, BIRC3, BIRC5, was consistently selected by LASSO-Cox, SVM, and RSF methods (**Figures 2G, H**). Correlation analysis further demonstrated strong positive associations among these 7 consensus genes, suggesting coordinated regulation within pyroptosis- and apoptosis-related networks (**Figure 2I**).

Then, we calculated PA score, based on the expression of the 7 consensus genes (the computational formula is provided in the methods). Patients were stratified into two groups based on the median of PA score and multivariate Cox regression analysis was carried out in TCGA-GBMLGG (**Supplementary Figure 3**). Expression profiling of these genes showed distinct patterns between patients with high and low PA scores and revealed close associations with established clinicopathological features, including WHO grade, IDH mutation status, MGMT promoter methylation, and treatment status (**Figure 2J**). Based on their PA scores, TCGA patients revealed a highly significant difference in overall survival (OS), with patients in the high-risk group exhibiting markedly poorer prognosis compared to those in the low-risk group (**Figure 2K**, Log-rank P < 0.0001). The three models established based on the 7 consensus genes all demonstrated satisfactory predictive ability for 1−year survival (**Figure 2L**). An independent external validation using the Chinese Glioma Genome Atlas (CGGA) cohort was used to assess the generalizability and clinical applicability of the model (**Figures 2M, N**, Log-rank P < 0.0001). Besides, we also validated the expression levels of PA.Sig using IHC staining, and the results indicated that the expression levels of Caspase-9 and GSDMD were upregulated in tumor cells than normal brain (**Figure 2O**).

### Association between PA.Sig and the immune microenvironment in glioma

**Figure 3:**
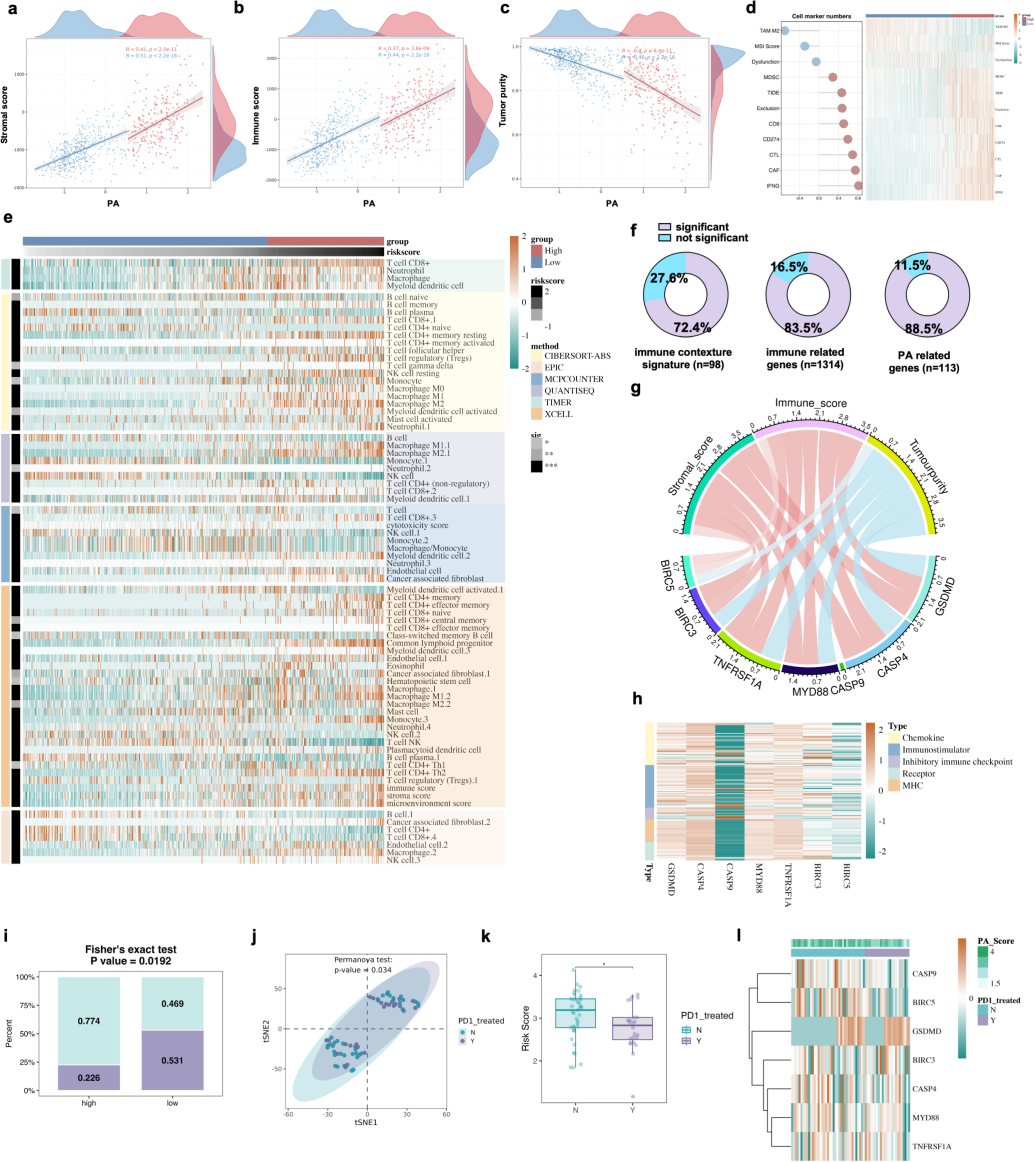
Association between PA.Sig and the immune microenvironment in glioma. **(A)** Scatter plots illustrate the correlation between PA score and Stromal score in PA-high and PA-low groups. **(B)** Scatter plots illustrate the correlation between PA score and ESTIMATE-derived ImmuneScore in PA-high and PA-low groups. **(C)** Scatter plots depict the correlation between PA score and ESTIMATE-inferred tumor purity in PA-high and PA-low groups. Spearman correlation coefficients and corresponding P values are indicated. **(D)** Association between PA score and TIDE-derived immune evasion signatures. Left panel: Lollipop plot showing Spearman correlation coefficients between PA score and TIDE-related immune signatures. Red dots indicate positive correlations, and blue dots indicate negative correlations. Right panel: Heatmap displaying z-score–scaled expression of TIDE-related markers across samples stratified by PA risk group. Samples are ordered according to PA score. Color intensity represents relative enrichment levels. **(E)** Heatmap illustrating significantly different immune cell populations (FDR < 0.05) identified by multiple deconvolution algorithms, including CIBERSORT-ABS, EPIC, MCPcounter, quanTIseq, TIMER, and xCell. Samples are ordered by increasing PA score. **(F)** Pie charts showing the proportions of signatures exhibiting significant difference between the two groups in 98 immune contexture signatures, 1,314 immune-related genes, and 113 PA-related genes, respectively. **(G)** Chord diagram showing Spearman correlations between the seven consensus genes and ESTIMATE-derived ImmuneScore, StromalScore, and tumor purity. **(H)** Heatmap showing Spearman correlation coefficients between the seven consensus genes and a curated panel of immunomodulators, including chemokines, immunostimulators, MHC molecules, receptors, and inhibitory immune checkpoints. **(I)** T-SNE visualization of PD-1–treated and untreated glioma samples. T-SNE plot based on batch-corrected and normalized expression profiles of PA-related genes from integrated immunotherapy cohorts. **(J)** Boxplot showing distribution of PA risk scores in PD-1–treated (Y) and untreated (N) samples. **(K)** Stacked bar plot showing the proportion of PD-1–treated and untreated samples in high- and low-risk groups. **(L)** Heatmap displaying z-score–scaled expression levels of the seven consensus genes across PD-1–treated and untreated samples.

To characterize the relationship between the PA score and tumor microenvironment composition, we analyzed ESTIMATE-derived stromal and immune metrics across TCGA gliomas. The PA score was positively correlated with both StromalScore and ImmuneScore in PA-high and PA-low groups (**Figures 3A, B**, all p < 0.001), indicating coordinated enrichment of stromal and immune components with increasing PA levels. In contrast, PA score showed a significant negative correlation with inferred tumor purity in both strata (PA-high: R = −0.40, P = 6.4 × 10^-^¹¹; PA-low: R = −0.48, P < 2.2 × 10^-^¹^6^) (**Figure 3C**). These findings collectively suggest that tumors with elevated PA scores are characterized by reduced tumor cell fraction and increased non-malignant cellular infiltration. TIDE-related functional indices revealed that PA score was positively correlated with multiple immune evasion–related signatures, including IFNG signature, cancer-associated fibroblasts (CAF), cytotoxic T lymphocyte (CTL) abundance, CD274 (PD-L1) expression, CD8 infiltration, TIDE score, T cell exclusion, and MDSC enrichment (**Figure 3D**). Conversely, negative correlations were observed for TAM M2 abundance, MSI score, and T cell dysfunction metrics. These findings suggest that tumors with elevated PA scores exhibit a complex immune landscape characterized by enhanced immune activation signals alongside increased markers of immune suppression and exclusion, indicating a potentially immunologically inflamed yet dysfunctional microenvironment. Using six computational algorithms, including CIBERSORT, EPIC, MCP-counter, TIMER, quanTIseq, and xCell, we comprehensively analyzed immune cell composition (**Figure 3E**). PA-high group exhibited enriched infiltration of multiple myeloid and lymphoid populations, including CD8^+^ T cells, macrophage subsets, dendritic cells, neutrophils, and cancer-associated fibroblasts, depending on the estimation framework. Importantly, these alterations were consistently observed across different computational platforms, underscoring the robustness of the immune remodeling pattern. Moreover, when considering 98 immunity contexture signatures, we observed that the majority of the genes were significantly associated with the risk score (**Figure 3F**). A similar trend was observed for the 1,314 immune-related genes and 113 PA-related genes.

To further elucidate the biological relevance of the 7 consensus genes, Spearman correlation analysis was performed between their expression levels and ESTIMATE-derived ImmuneScore, StromalScore, and tumor purity. The chord diagram illustrates coordinated associations between the 7 consensus genes (CASP4, CASP9, MYD88, TNFRSF1A, BIRC3, BIRC5, and GSDMD) and tumor microenvironment metrics (**Figure 3G**). Most genes exhibited positive correlations with ImmuneScore and StromalScore, while demonstrating inverse associations with tumor purity, consistent with the overall PA score pattern. Then we evaluated their correlations with a curated panel of immunomodulators, including chemokines, immunostimulators, MHC molecules, receptors, and inhibitory immune checkpoints (**Figure 3H**). The seven consensus genes generally showed positive associations with multiple immune-related molecules, indicating coordinated interactions with the immune microenvironment. In contrast, CASP9 displayed a distinct pattern with predominantly negative correlations. This opposite trend suggests potential functional heterogeneity among the consensus genes in their interaction with tumor immunity. Procrustes analysis demonstrated a significant concordance between PA.Sig gene expression and the seven-step cancer-immunity cycle (Mantel r = 0.7673, P = 0.001; Procrustes M² = 0.344, P = 0.001; **Supplementary Figure 4A**). Stepwise Mantel tests further indicated that PA.Sig was most strongly correlated with Step 4 (trafficking of immune cells, r = 0.00605, P = 0.002) and Step 6 (recognition of cancer cells, r = 0.00470, P = 0.013; **Supplementary Figure 4B**). In merged immunotherapy cohorts (GSE121810 and PRJNA482620), PA.Sig high-risk patients showed significantly lower response rates to anti-PD1 therapy (Fisher’s exact test, P = 0.034; **Figure 3I**). t-SNE visualization based on PA.Sig gene expression clearly separated responders from non-responders (PERMANOVA, P = 0.034; **Figure 3J**). Risk scores were significantly higher in non-responders (Wilcoxon test, P < 0.05; **Figure 3K**), and a heatmap of the seven consensus genes further confirmed distinct expression patterns between response groups (**Figure 3L**).

### Distinct genomic alterations and enriched biological pathway in PA.Sig risk groups

**Figure 4:**
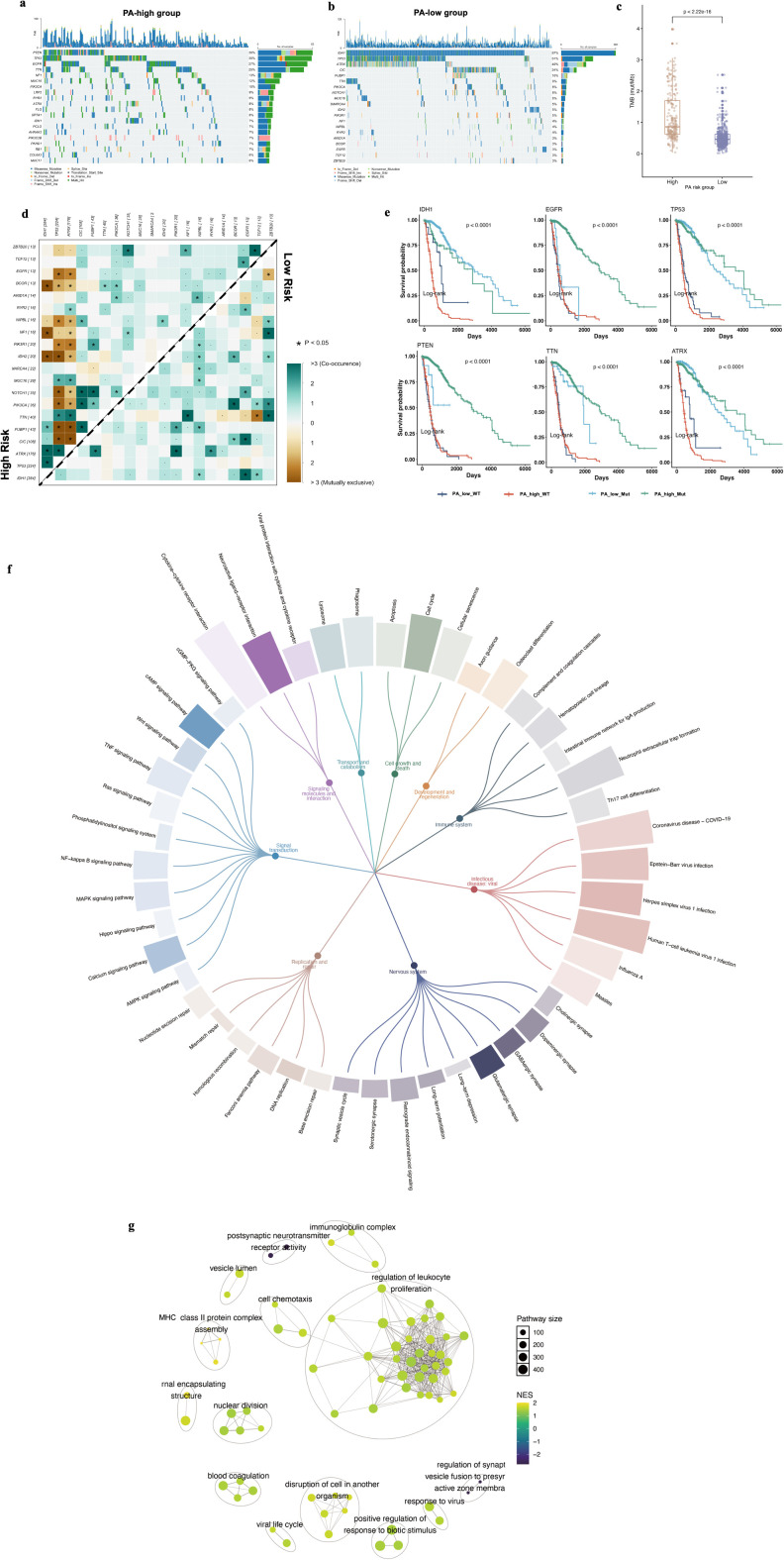
Distinct genomic alterations and enriched biological pathway in PA.Sig risk groups. **(A, B)** Oncoplot of the top 20 most frequently mutated genes in the PA-high **(A)** and PA-low **(B)** group. **(C)** Comparison of tumor mutation burden (TMB) between PA-high and PA-low groups. **(D)** Somatic interaction analysis in PA-defined subgroups. **(E)** Kaplan–Meier survival analysis stratified by PA risk group and driver gene mutation status. **(F)** Differentially expressed genes between high- and low-risk groups were subjected to KEGG enrichment analysis. Pathways are grouped by functional subclasses and visualized as a circular hierarchical network. Branches represent subclass–pathway relationships, and bar lengths indicate gene counts within each enriched pathway. **(G)** Gene set enrichment analysis was performed using GO terms and visualized as a network. Nodes represent enriched GO terms, with node size proportional to pathway size. Color indicates normalized enrichment score (NES). Edges denote functional similarity between terms. Distinct functional clusters highlight immune activation, cell cycle regulation, and stress-response pathways.

To characterize genomic alterations associated with the PA signature, mutation profiling was performed in the TCGA-GBMLGG cohort. The overall mutational landscape was dominated by single nucleotide variants, with missense mutations as the most frequent classification. The most recurrently mutated genes included IDH1, TP53, and ATRX, followed by PTEN, EGFR, TTN, and CIC (**Supplementary Figure 5A**). Stratification by PA risk score revealed distinct mutational patterns. The low-risk group was enriched for canonical IDH-mutant glioma alterations, including IDH1 (87%), TP53 (~51%), ATRX (~40%), CIC (~24%), and FUBP1 (~10%) (**Figure 4A**). In contrast, the high-risk group exhibited predominant mutations in PTEN and TP53 (~30%), EGFR (~27%), and NF1 (~13%), consistent with IDH–wild-type and aggressive glioma features (**Figure 4B**). IDH1 mutations were markedly less frequent in the high-risk cohort. Moreover, tumor mutation burden was significantly elevated in high-risk patients (**Figure 4C**, P < 2.22 × 10^-^¹^6^).

Somatic interaction analysis further demonstrated distinct genetic architectures. The low-risk subgroup displayed a tightly connected co-occurrence network centered on IDH1, ATRX, CIC, and FUBP1, whereas the high-risk group showed significant mutual exclusivity among EGFR, PTEN, and NF1, suggesting alternative activation of RTK/PI3K signaling pathways (**Figure 4D**). Kaplan–Meier analyses integrating mutation status and PA risk demonstrated significant survival differences across subgroups for multiple driver genes, including IDH1, EGFR, TP53, PTEN, TTN, and ATRX (**Figure 4E**, all P < 0.0001). High-risk patients exhibited inferior survival regardless of mutation status, whereas low-risk patients with IDH1 or ATRX mutations showed the most favorable outcomes, indicating that the prognostic value of the PA signature extends beyond individual driver alterations. Transcriptomic pathway analysis further supported these distinctions. KEGG enrichment revealed that high-risk tumors were associated with pathways related to signal transduction, cell growth and death, replication and repair, and immune activation, whereas low-risk tumors showed enrichment in developmental and metabolic processes (**Figure 4F**). GO-based GSEA identified coordinated modules involving immune regulation, cell cycle progression, and stress-response pathways, reinforcing the biological divergence between PA-defined risk groups (**Figure 4G**).

### Pyroptosis–apoptosis score delineates malignant cell state heterogeneity and differentiation trajectories

**Figure 5:**
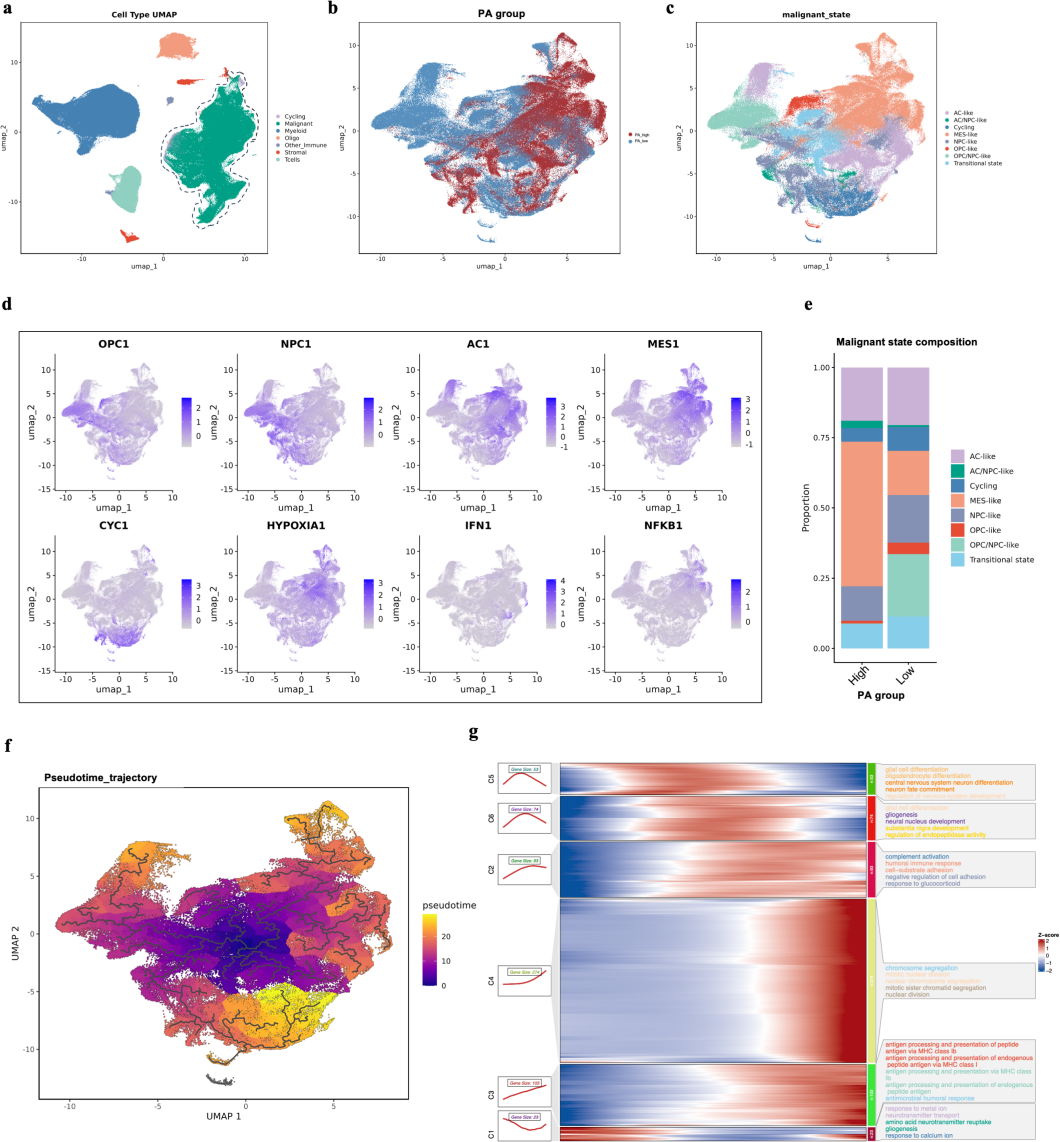
Pyroptosis–apoptosis (PA) score delineates malignant cell state heterogeneity and differentiation trajectories. **(A)** UMAP projection of all cells colored by annotated cell types. Major cell populations include malignant cells, myeloid cells, T cells, oligodendrocytes, stromal cells, cycling cells, and other immune cells. Malignant cells were marked with dotted lines **(B)** UMAP projection of malignant cells colored by PA group assignment. Cells were classified into PA-high and PA-low groups based on the sample-level PA score. **(C)** UMAP projection of malignant cells colored by annotated malignant cell states, including AC-like, NPC-like, OPC-like, MES-like, cycling, and transitional states, based on state-specific gene expression programs. **(D)** Feature plots showing the expression or activity scores of OPC-like, NPC-like, AC-like, MES-like, cycling, hypoxia, interferon, and NF-κB–related gene programs projected onto the UMAP embedding of malignant cells. Color intensity indicates relative signature enrichment at the single-cell level. **(E)** Stacked bar plots showing the proportional distribution of malignant transcriptional states in PA-high and PA-low malignant cell populations. **(F)** Pseudotime trajectory inferred from malignant cells and overlaid on the UMAP embedding. Cells are colored by pseudotime values, with darker colors indicating earlier pseudotime and lighter colors indicating later pseudotime. Black lines represent the inferred trajectory backbone. **(G)** Heatmap showing pseudotime-ordered gene expression patterns clustered into distinct modules. Representative Gene Ontology biological processes enriched in each module are indicated on the right. Gene expression values are Z-score normalized.

To investigate how coordinated activation of pyroptosis and apoptosis programs shapes tumor-intrinsic heterogeneity, we analyzed annotated scRNA-seq data from glioma patients (GSE274546 and SCP cohorts) (**Figure 5A**). Pseudo-bulk expression profiles were generated by aggregating malignant cells at the biosample level, followed by TPM normalization and log-transformation. The PA score was subsequently projected back to single cells, allowing stratification of malignant cells into PA-high and PA-low groups (**Figure 5B**). Malignant cells were further classified into eight states using lineage-specific gene signatures: AC-like, AC/MES-like, Cycling, MES-like, NPC-like, OPC-like, OPC/NPC-like, and Transitional (**Figure 5C**). Lineage-associated programs, including OPC-like, NPC-like, and AC-like signatures, exhibited distinct yet partially overlapping spatial distributions, consistent with the previously identified malignant cell states (**Figure 5D**). In contrast, MES-like signatures showed a broader and more diffuse distribution across the UMAP space, suggesting activation across multiple malignant subpopulations. In addition, functional programs related to cell cycling, hypoxia, interferon signaling, and NF-κB activation displayed heterogeneous and regionally enriched patterns. Notably, hypoxia- and inflammatory-related signatures were preferentially enriched in specific malignant regions, indicating the coexistence of diverse stress-adaptive transcriptional programs within the tumor cell population (**Figure 5D**). Importantly, PA-high and PA-low malignant cells displayed markedly different state compositions: PA-high tumors were enriched for MES-like populations, whereas PA-low tumors showed a higher proportion of OPC-like and NPC-like states, suggesting divergent differentiation trajectories associated with PA activity (**Figure 5E**). The trajectory analysis revealed a continuous and branched pseudo temporal structure, with cells at early pseudotime occupying a central region of the UMAP space, while cells at later pseudotime were distributed toward multiple peripheral regions. This pattern suggests progressive divergence of malignant cells into distinct transcriptional programs during tumor evolution. Notably, the inferred pseudotime progression recapitulated the spatial organization of malignant cell states and functional programs observed previously, supporting a dynamic transition from lineage-associated states toward mesenchymal-like and stress-adaptive phenotypes (**Figure 5F**). To characterize transcriptional dynamics along malignant cell evolution, genes were clustered based on their expression patterns along pseudotime, resulting in multiple pseudotime-dependent gene modules (**Figure 5G**). Early pseudotime modules were predominantly enriched for neural lineage–associated biological processes, including gliogenesis, oligodendrocyte differentiation, neural development, and neuron fate commitment, indicating a lineage-associated transcriptional program at early stages. As pseudotime progressed, intermediate modules showed enrichment of cell adhesion, humoral immune response, and complement activation, suggesting a gradual shift toward microenvironment interaction and immune-related programs. In contrast, late pseudotime modules were characterized by strong activation of cell cycle–related processes, such as chromosome segregation, mitotic nuclear division, and nuclear division, as well as antigen processing and presentation pathways, reflecting proliferative and immunologically active malignant states at advanced stages of pseudotime. Genes associated with mesenchymal-like and stress-adaptive programs, including APOE and FAM111B, showed progressive upregulation toward late pseudotime, whereas LIF, a cytokine involved in inflammatory signaling, displayed a transient peak at intermediate-to-late pseudotime (**Supplementary Figure 6A**). In contrast, neuronal and lineage-associated genes, such as GRIA2 and NRXN1, exhibited higher expression at early pseudotime followed by gradual downregulation along the trajectory, consistent with attenuation of neuronal identity during malignant progression (**Supplementary Figure 6B**). Together, these gene-level dynamics provide molecular support for the inferred pseudotime trajectory.

### PA-associated malignant programs reshape T cell states and death-related intercellular communication

**Figure 6:**
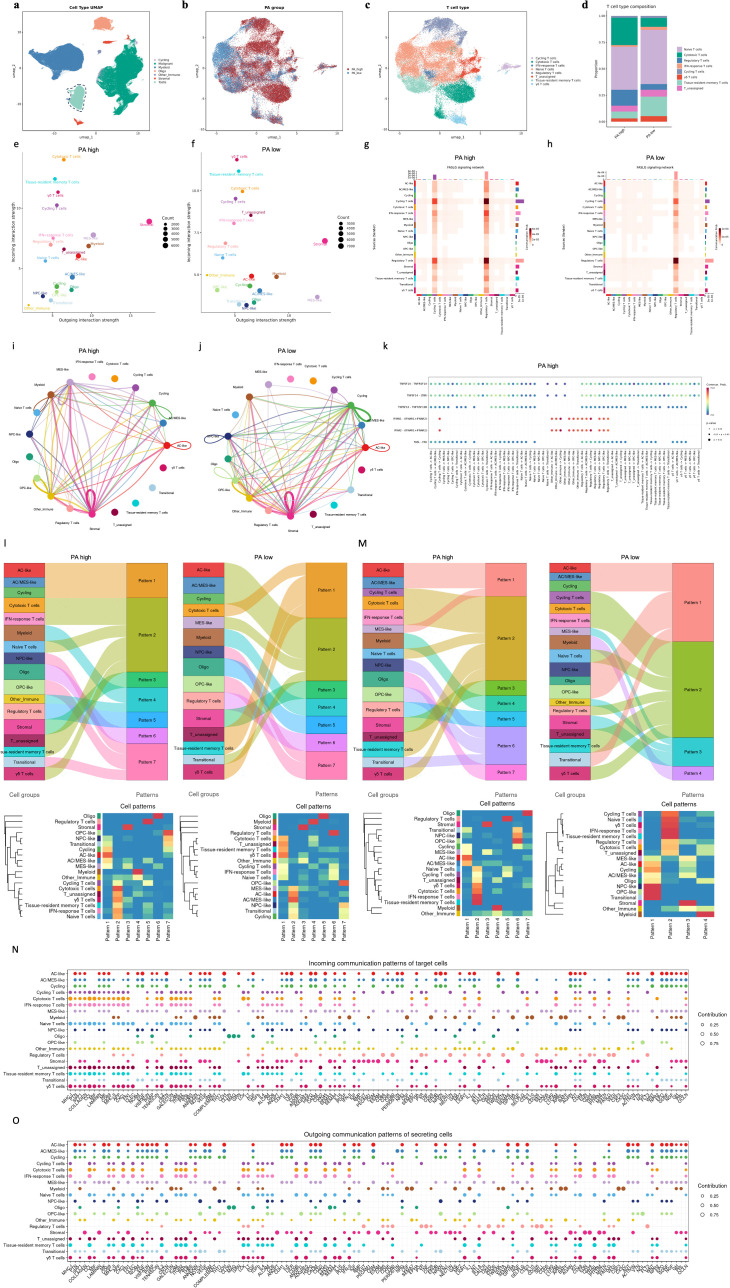
PA-associated malignant programs reshape T cell states and death-related intercellular communication. **(A)** UMAP projection of all cells colored by annotated cell types. Major cell populations include malignant cells, myeloid cells, T cells, oligodendrocytes, stromal cells, cycling cells, and other immune cells. T cells was marked with dotted lines. **(B)** UMAP projection of T cells colored by PA group assignment. Cells were classified into PA-high and PA-low groups based on the sample-level PA score. **(C)** UMAP projection of T cells colored by annotated T cell subtypes, including naïve, cytotoxic, regulatory, tissue-resident memory, IFN-response, cycling, and γδ T cells. **(D)** Stacked bar plots showing the proportional distribution of annotated T cell subtypes across groups. **(E, F)** Scatter plots showing the outgoing (x-axis) and incoming (y-axis) interaction strength of each cell population inferred by CellChat in PA-high **(E)** and PA-low **(F)** tumors. Dot size represents the number of cells in each population. **(G, H)** Heatmaps showing inferred FASLG signaling interactions between source (rows) and target (columns) cell populations in PA-high **(G)** and PA-low **(H)** tumors. Color intensity represents communication probability as inferred by CellChat. **(I, J)** Circle plots showing CellChat-inferred IFNA2–IFNAR1/IFNAR2 signaling interactions among malignant, immune, and stromal cell populations in PA-high **(I)** and PA-low **(J)** tumors. Edge width represents interaction strength. **(K)** Dot plot showing CellChat-inferred ligand–receptor interactions in PA-high tumors. Dot color indicates communication probability, and dot size reflects statistical significance. Incoming **(L)** and outgoing **(M)** communication patterns differ between PA-high and PA-low tumors. Incoming **(N)** and outgoing **(O)** communication patterns of target cells in PA-high tumors.

Given the intimate crosstalk between malignant cells and the immune microenvironment, we next examined how PA activity influences T cell composition and intercellular communication. Extracted T cells was annotated by calculated Pseudo-bulk expression profiles (**Figures 6A, B**). Then these T cells were re-clustered and annotated based on canonical transcriptional programs, yielding naïve T cells, cytotoxic T cells, regulatory T cells, tissue-resident memory T cells, IFN-response T cells, cycling T cells, and γδ T cells (**Figure 6C**). Projection of the PA grouping onto T cells revealed pronounced differences in T cell state distributions between PA-high and PA-low tumors. PA-high tumors exhibited an increased abundance of regulatory T cells and cytotoxic T cells, whereas PA-low tumors were relatively enriched for naïve and tissue-resident memory T cells (**Figure 6D**). Comparison of the aggregated communication networks revealed a marked decrease in both the number and overall strength of inferred cell–cell interactions in the PA-high group (**Supplementary Figure 7A**). Centrality analysis found little signal derived from malignant cells. In contrast, immune populations, including multiple T cell subsets, primarily functioned as signal receivers in PA-high tumors (**Figure 6E**). In PA-low tumors, MES-like malignant cells acted as dominant outgoing signaling hubs (**Figure 6F**). This shift in signaling hierarchy suggests that PA-high malignant cells inactively orchestrate microenvironmental interactions, potentially affecting immune cell activation. Focusing on apoptosis-associated pathways, including FASLG, TRAIL, LIGHT, and TWEAK, very few signals are emitted by T cell hubs and received by malignant cells in both PA-low and PA-high groups (**Figures 6G, H**, **Supplementary Figure 7B**). In pyroptosis-related inflammatory pathways, including IL1, IFN-I, and IFN-II signaling, although a large number of signals were released by malignant cells in both the PA-low group and the PA-high group, they rarely directly linked to T cells, especially IFN-response T cells, cytotoxic T cells and γδ T cells (**Figures 6I, J**, **Supplementary Figure 7C**). To dissect the molecular basis of PA-associated signaling, we quantified the contribution of individual ligand–receptor pairs. Key ligand–receptor pairs comprising FASL–FAS, TNFSF12–TNFRSF12A (TWEAK–FN14), IFNG–IFNGR1/2, and IL1A/B–IL1R1/IL1RAP, underscoring the convergence of apoptotic and pyroptotic signaling on immune-targeted communication accounted for a substantial proportion of the total communication probability in PA-high tumors (**Figure 6K**). In contrast, these ligand–receptor interactions contributed minimally to the communication landscape in PA-low tumors (**Supplementary Figure 7E**). To capture coordinated signaling programs beyond individual pathways, we analyzed incoming and outgoing communication patterns inferred by CellChat (**Supplementary Figure 7D**). In PA-high tumors, incoming signaling was concentrated within malignant and stromal compartments, with AC-like, AC/MES-like, MES-like, and stromal cells showing strong enrichment for immune-derived inputs, including interferon-, TNF-, FAS-, and antigen presentation–related pathways (**Figure 6L**). This pattern indicates that advanced malignant states serve as major integrators of immune-mediated signals. In parallel, outgoing signaling patterns in PA-high tumors were dominated by malignant and stromal cells, which exhibited broad and high-contribution output across immune- and inflammation-related pathways (**Figure 6M**). In contrast, PA-low tumors displayed a more heterogeneous and redistributed organization of both incoming and outgoing signaling patterns, involving a wider range of immune cell subsets and reduced malignant signaling dominance (**Figures 6L, M**). In PA-high tumors, malignant and stromal cells dominated outgoing signaling across interferon, TNF, FAS, and cytokine-related pathways, indicating tumor-centered signal propagation. Concurrently, malignant populations were preferentially enriched for incoming inflammatory and immune-associated inputs, positioning them as major integrators of microenvironmental signals (**Figures 6N, O**). In contrast, PA-low tumors displayed a more distributed organization of both outgoing and incoming signaling, with greater immune cell participation and reduced malignant dominance (**Supplementary Figures 7F, G**). These coordinated differences suggest that PA-high tumors operate within a self-reinforcing communication architecture centered on advanced malignant states. Collectively, PA status defines a shift from malignant-driven signaling dominance to a more balanced tumor–immune communication topology.

## Discussion

In this study, we developed and validated a pyroptosis–apoptosis–associated gene signature (PA.Sig) that robustly stratifies glioma patients into clinically and biologically distinct subgroups. Across independent cohorts, PA.Sig demonstrated strong prognostic value and remained informative beyond established clinicopathologic factors, supporting its utility as a risk stratification tool in glioma.

A key finding of this work is that PA-high tumors exhibit an immune-enriched yet functionally suppressed microenvironment. Although PA score positively correlated with immune and stromal infiltration, PA-high gliomas were characterized by elevated TIDE scores, increased immune exclusion signatures, and enrichment of immunosuppressive cell populations. These data suggest that activation of death-associated inflammatory pathways does not necessarily translate into effective antitumor immunity. Consistently, in independent anti–PD-1 cohorts, PA-high patients showed inferior therapeutic responses, indicating that PA.Sig may serve as a predictive biomarker for immunotherapy efficacy.

At the genomic level, PA-high tumors were enriched for IDH–wildtype–associated alterations, including EGFR and PTEN mutations, and displayed increased tumor mutation burden, consistent with a more aggressive molecular phenotype. In contrast, PA-low tumors were dominated by canonical IDH1–ATRX–CIC co-occurrence patterns typical of lower-grade glioma. These findings suggest that PA activation is embedded within broader oncogenic programs linked to genomic instability and therapeutic resistance.

Single-cell analyses further revealed that PA-high malignant cells preferentially adopted mesenchymal-like and proliferative states, whereas PA-low tumors retained lineage-associated programs. Pseudotime modeling supported a shift toward stress-adaptive and inflammatory phenotypes along tumor evolution, indicating that coordinated pyroptosis–apoptosis signaling may reflect advanced malignant cell states rather than terminal cell death alone.

Cell–cell communication analyses demonstrated a tumor-centered signaling architecture in PA-high gliomas, with malignant compartments dominating inflammatory and apoptosis-related pathways. Despite abundant cytokine signaling, effective cytotoxic communication toward T cell subsets appeared limited, suggesting a decoupling between immune activation signals and immune effector function. This may partially explain the reduced immunotherapy responsiveness observed in PA-high patients.

Functionally, temozolomide treatment induced concurrent activation of apoptotic and pyroptotic pathways in glioma cells, accompanied by ROS accumulation and mitochondrial dysfunction. Given the heterogeneous expression and activation profiles of various caspases in glioma cells, TMZ likely engages a dynamic and context-dependent caspase network rather than a uniform linear cascade, contributing to the mixed apoptotic/pyroptotic phenotype. Genetic perturbation of GSDMD and CASP4 attenuated lytic cell death, confirming that PA-related genes actively contribute to chemotherapy-induced hybrid death phenotypes. These findings provide mechanistic support linking the PA signature to therapeutic response.

Several limitations should be acknowledged. Most transcriptomic analyses were retrospective, and functional validation was performed in a single cell line. Prospective validation and *in vivo* modeling are warranted to confirm clinical applicability and further elucidate mechanistic interactions between pyroptosis and apoptosis in glioma. The expression of 7 consensus genes in clinical sample wasn’t totally confirmed in this study. The remaining genes need to be validated in future works.

In summary, PA.Sig defines a high-risk glioma subtype characterized by genomic instability, mesenchymal transition, immune enrichment with functional suppression, and reduced immunotherapy responsiveness. Beyond its prognostic value, PA.Sig provides a biologically grounded framework that may inform therapeutic decision-making and guide rational combination strategies integrating chemotherapy and immunomodulatory approaches.

## Data Availability

The datasets presented in this study can be found in online repositories. The names of the repository/repositories and accession number(s) can be found in the article/**Supplementary Material**.
