# Supplementary figures and images for: A cell death program–based tumor signature stratifies prognosis, immune landscape, and therapeutic response in glioma
Source: Front Oncol. 2026 May 21;16:1824504. doi: 10.3389/fonc.2026.1824504 (PMC13233278; doi:10.3389/fonc.2026.1824504)

**a**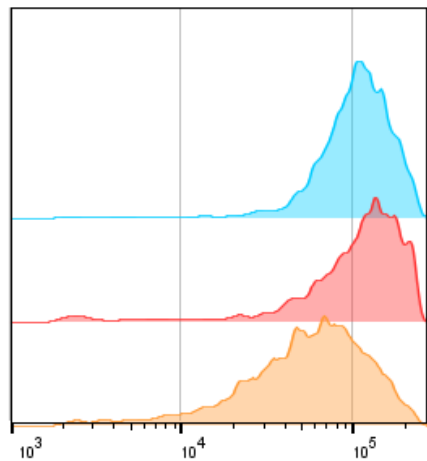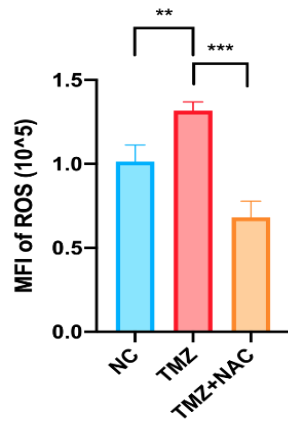**b**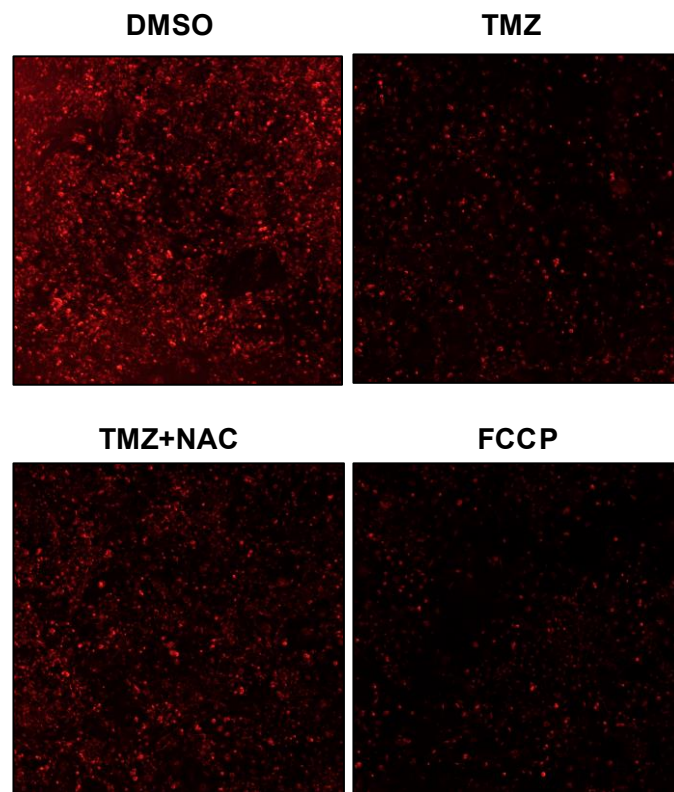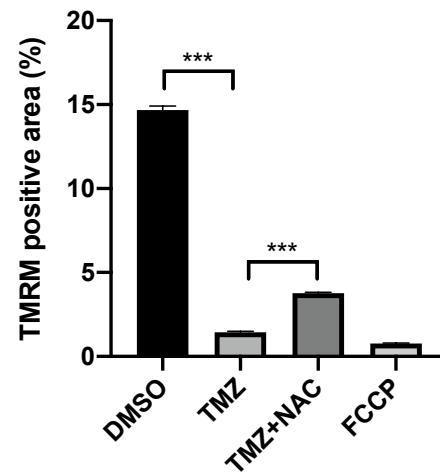

Supplement: Supplementary file 1 [file DataSheet1.pdf]

Top 10 of GO terms Enrichment

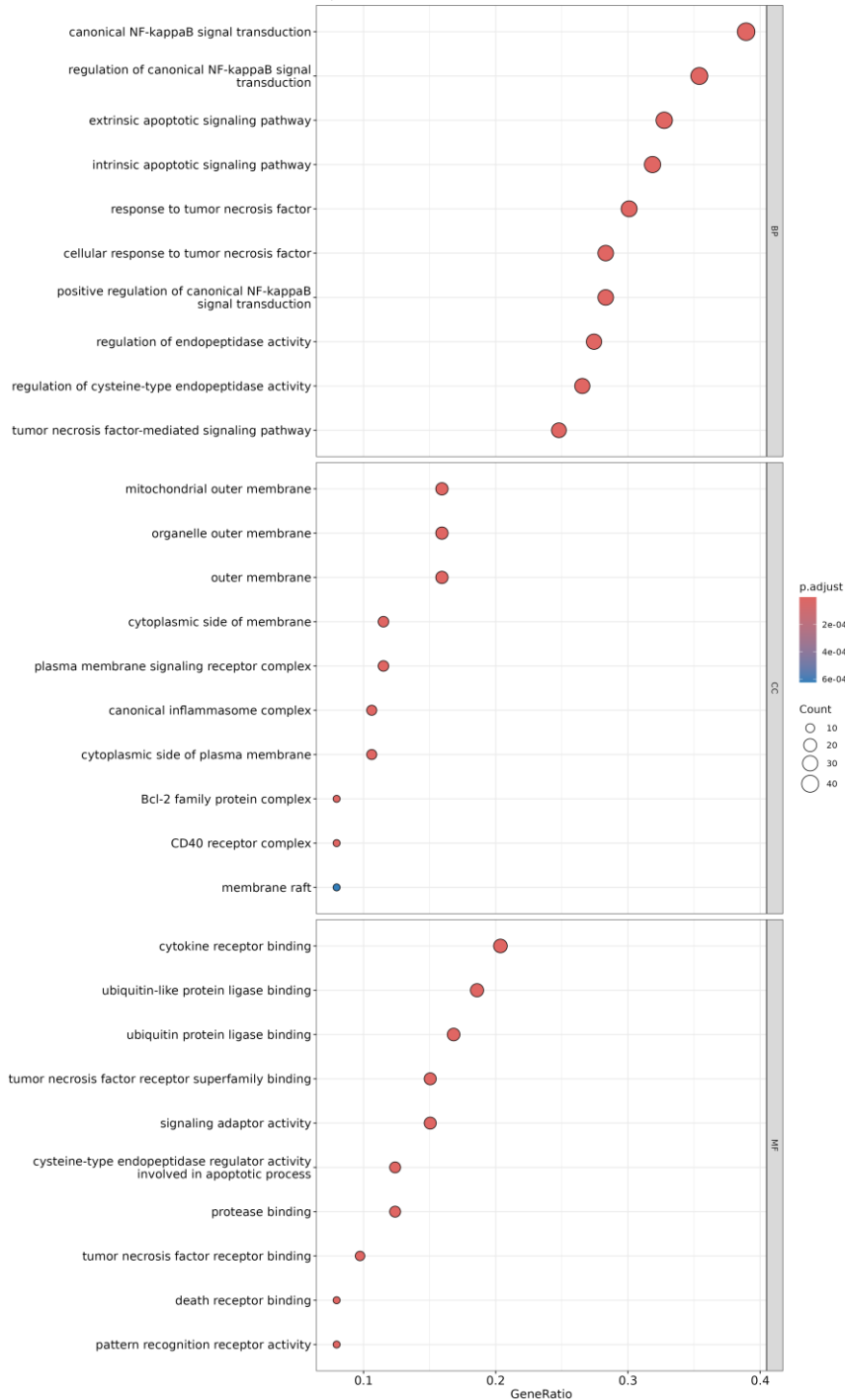

Reactome Pathway Enrichment

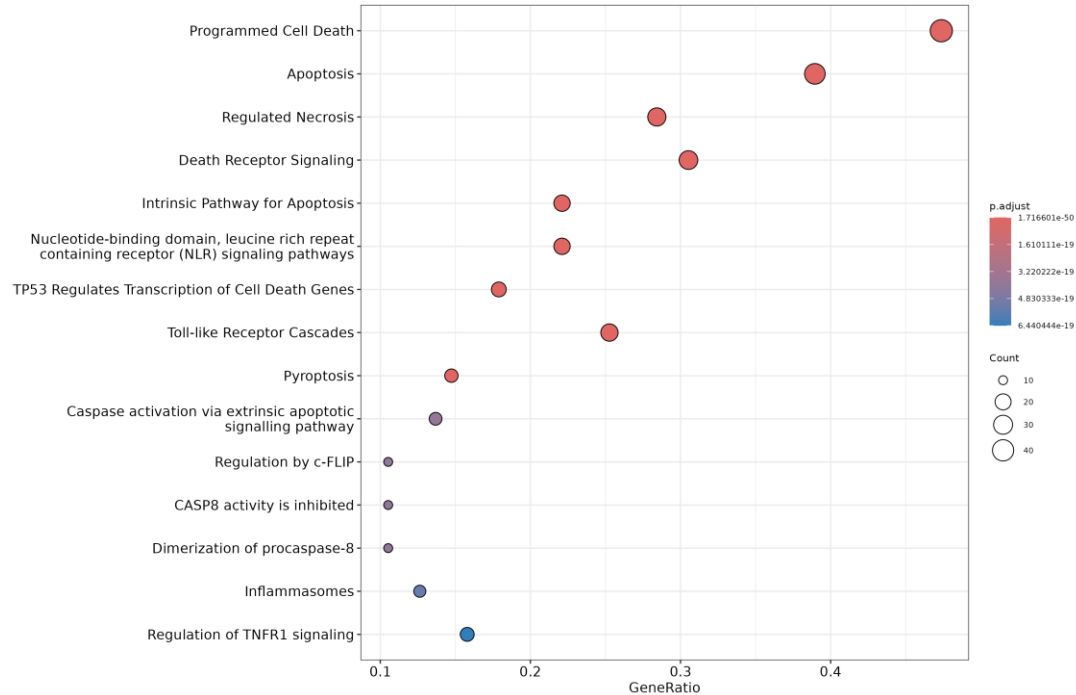

KEGG Pathway Enrichment

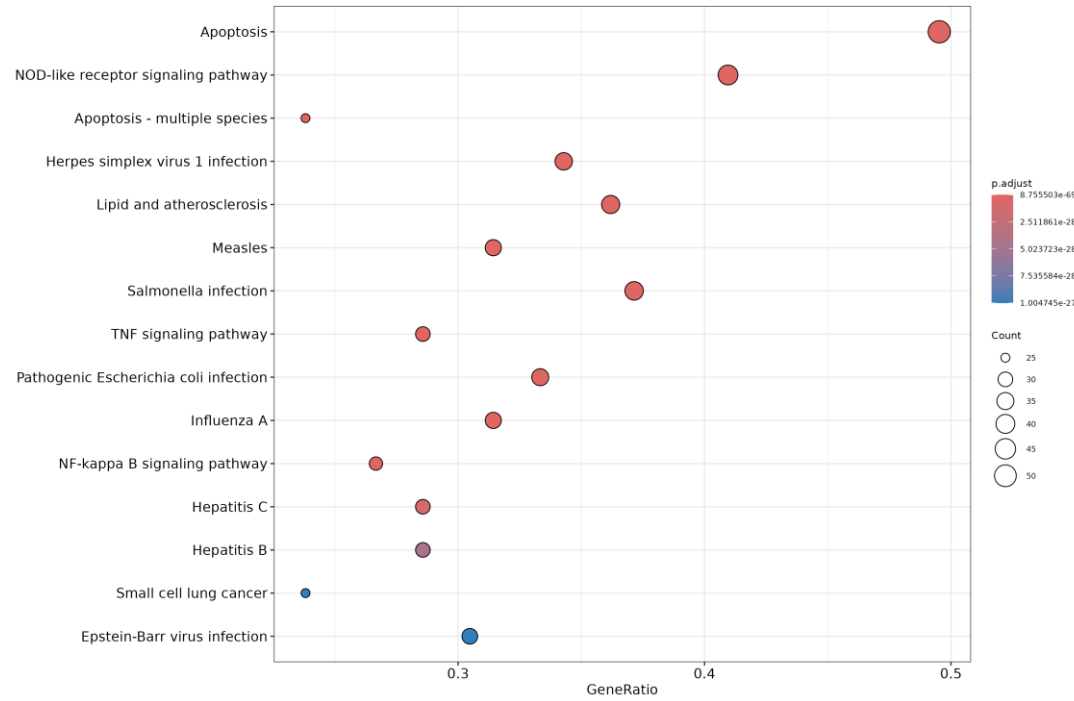

Supplement: Supplementary file 2 [file DataSheet2.pdf]

**a**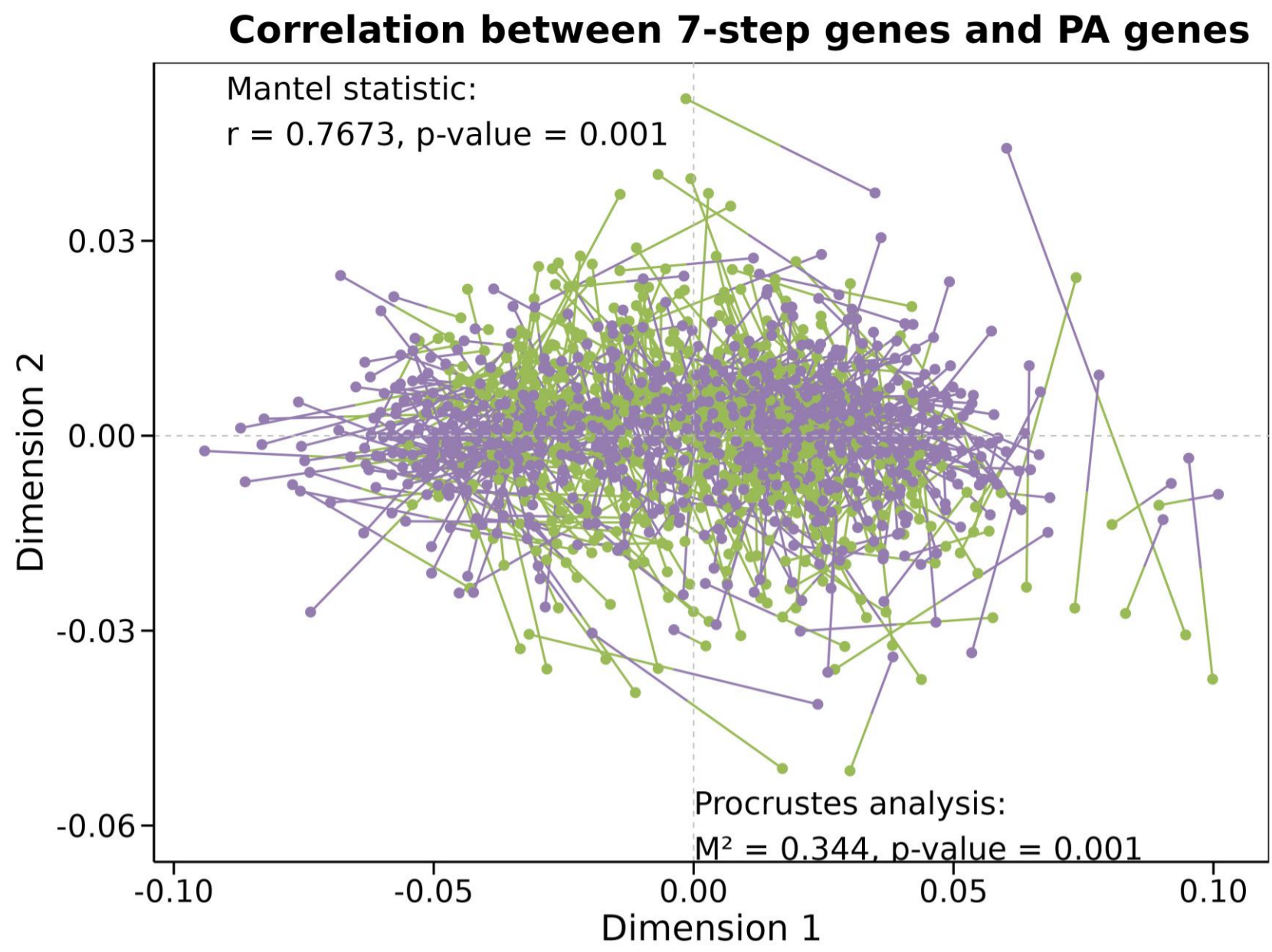**b**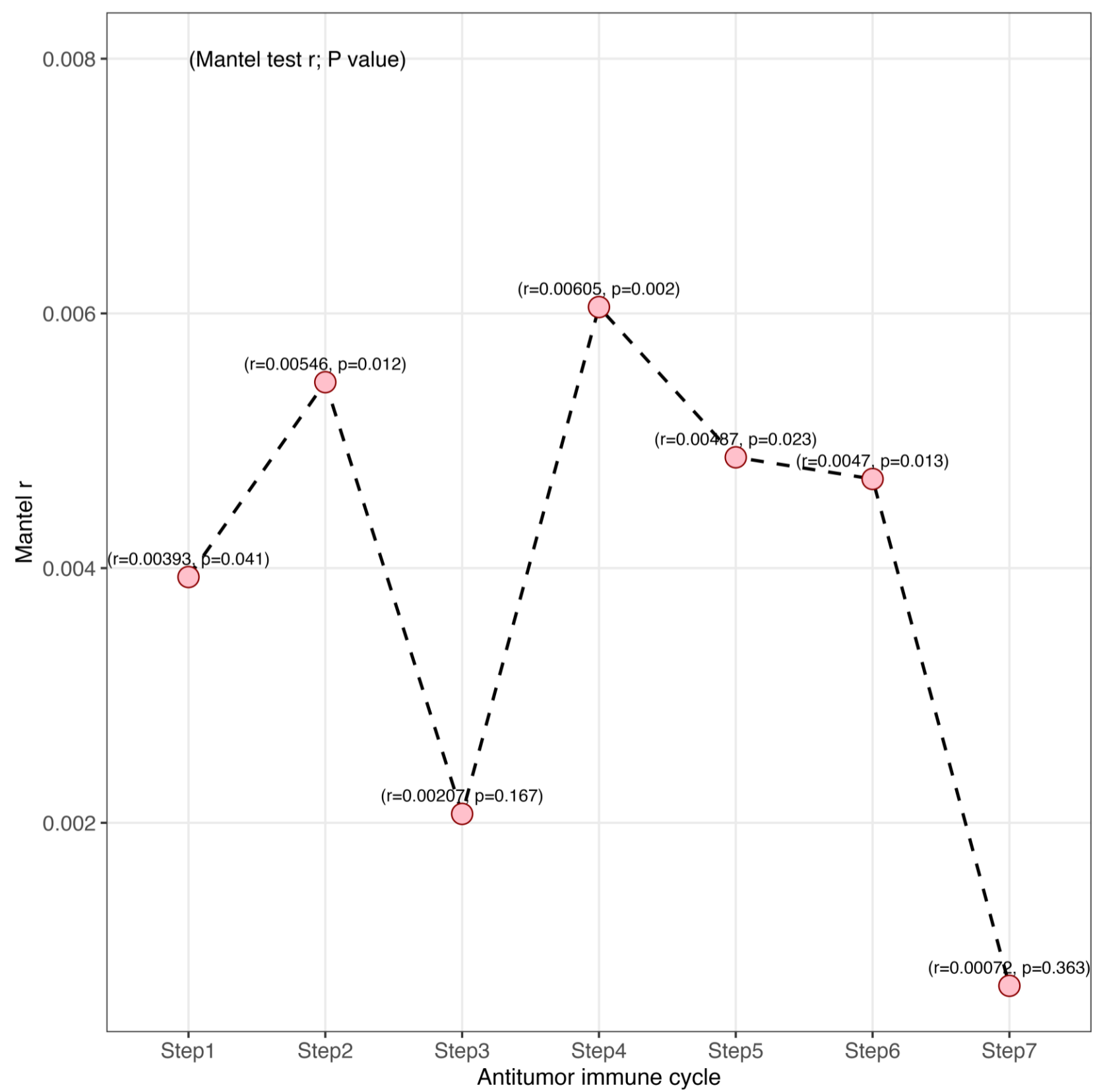

Supplement: Supplementary file 4 [file DataSheet4.pdf]

**a**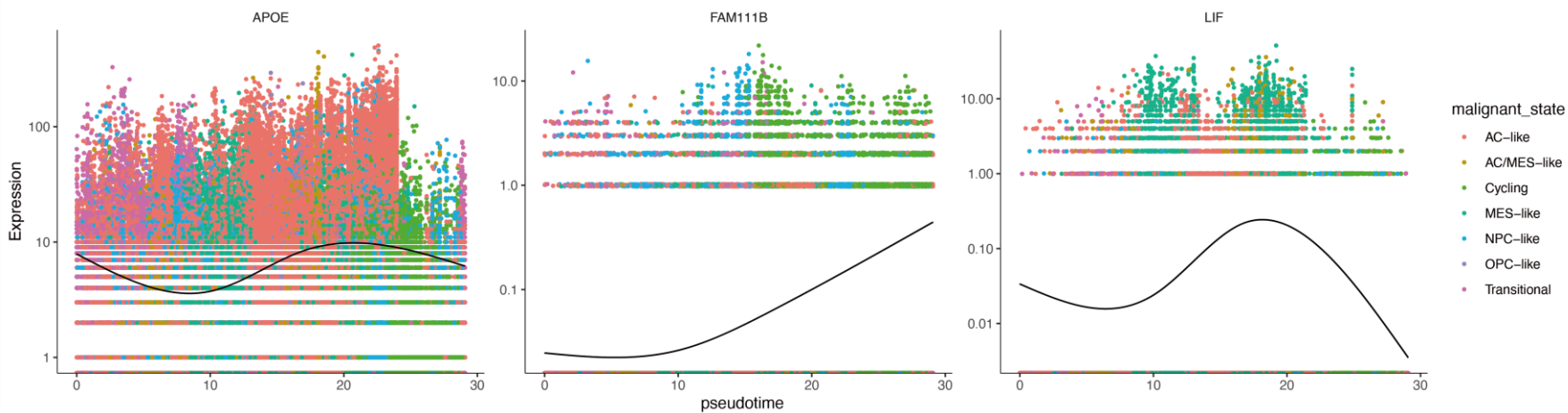**b**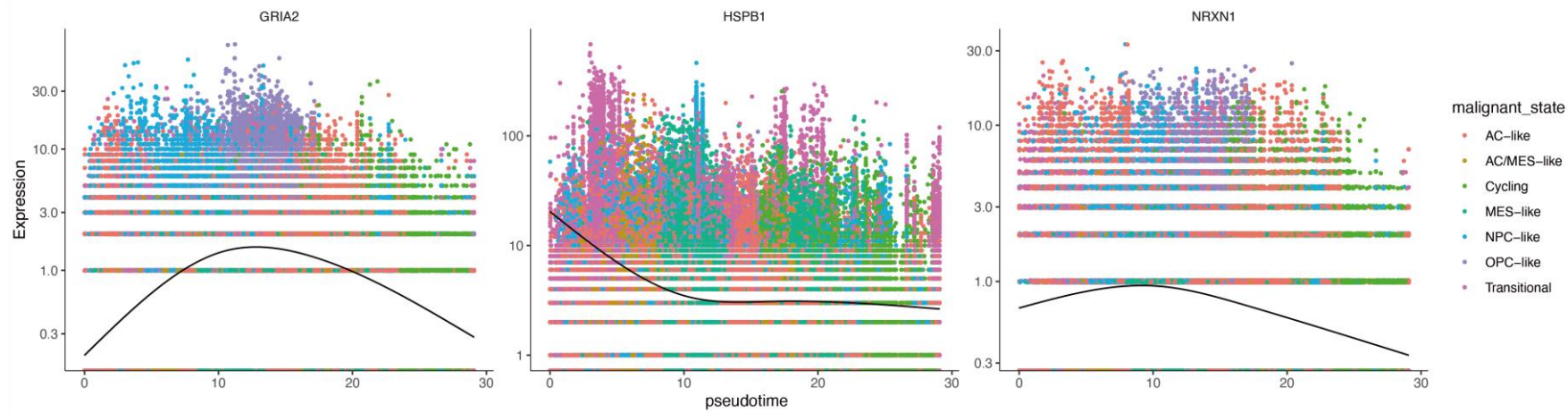

Supplement: Supplementary file 6 [file DataSheet6.pdf]

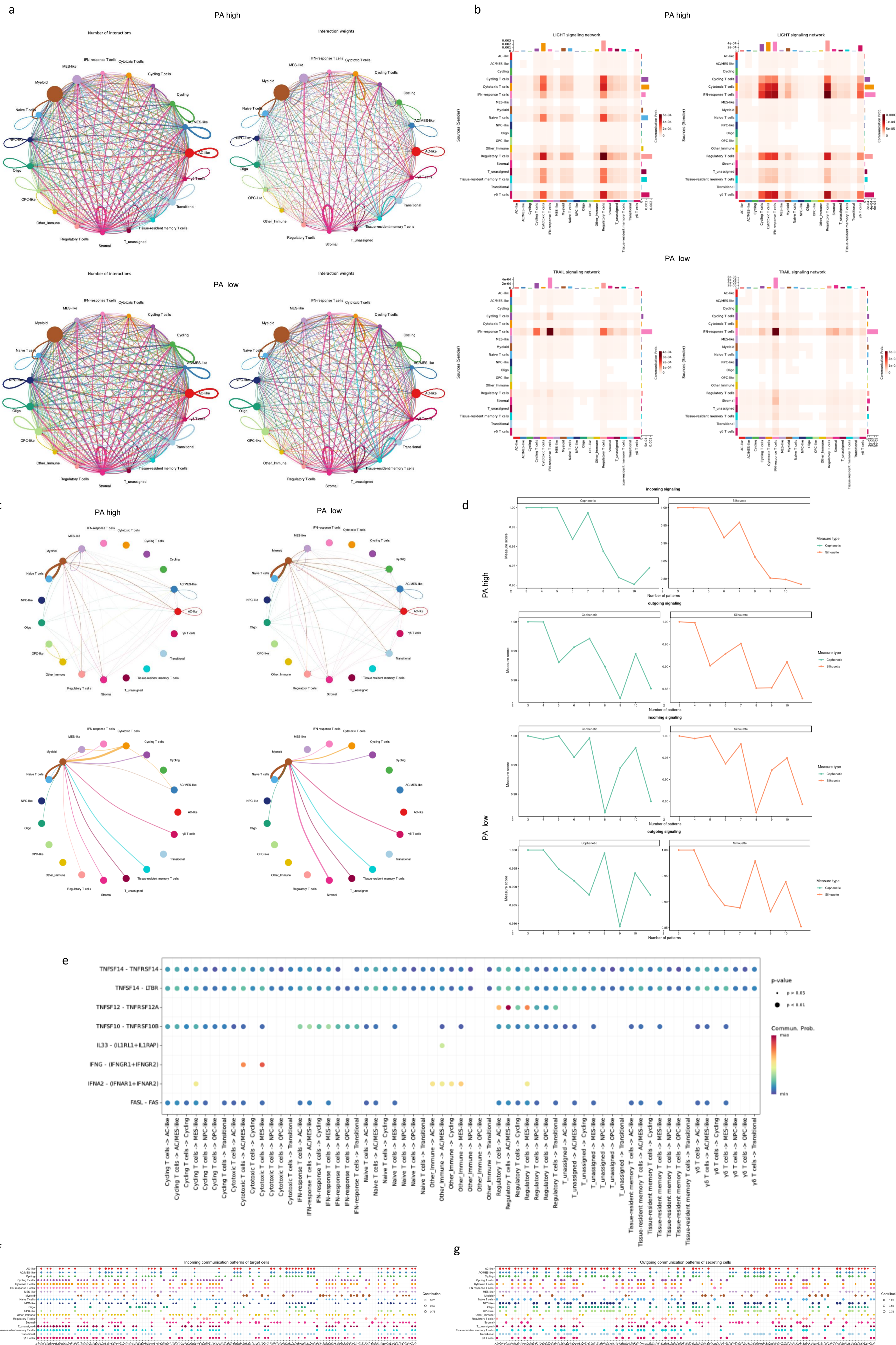

Supplement: Supplementary file 7 [file DataSheet7.pdf]
