# Supplementary material for: A cell death program–based tumor signature stratifies prognosis, immune landscape, and therapeutic response in glioma
Source: Front Oncol. 2026 May 21;16:1824504. doi: 10.3389/fonc.2026.1824504 (PMC13233278; doi:10.3389/fonc.2026.1824504)

Multivariate Cox Regression Analysis
Forest Plot

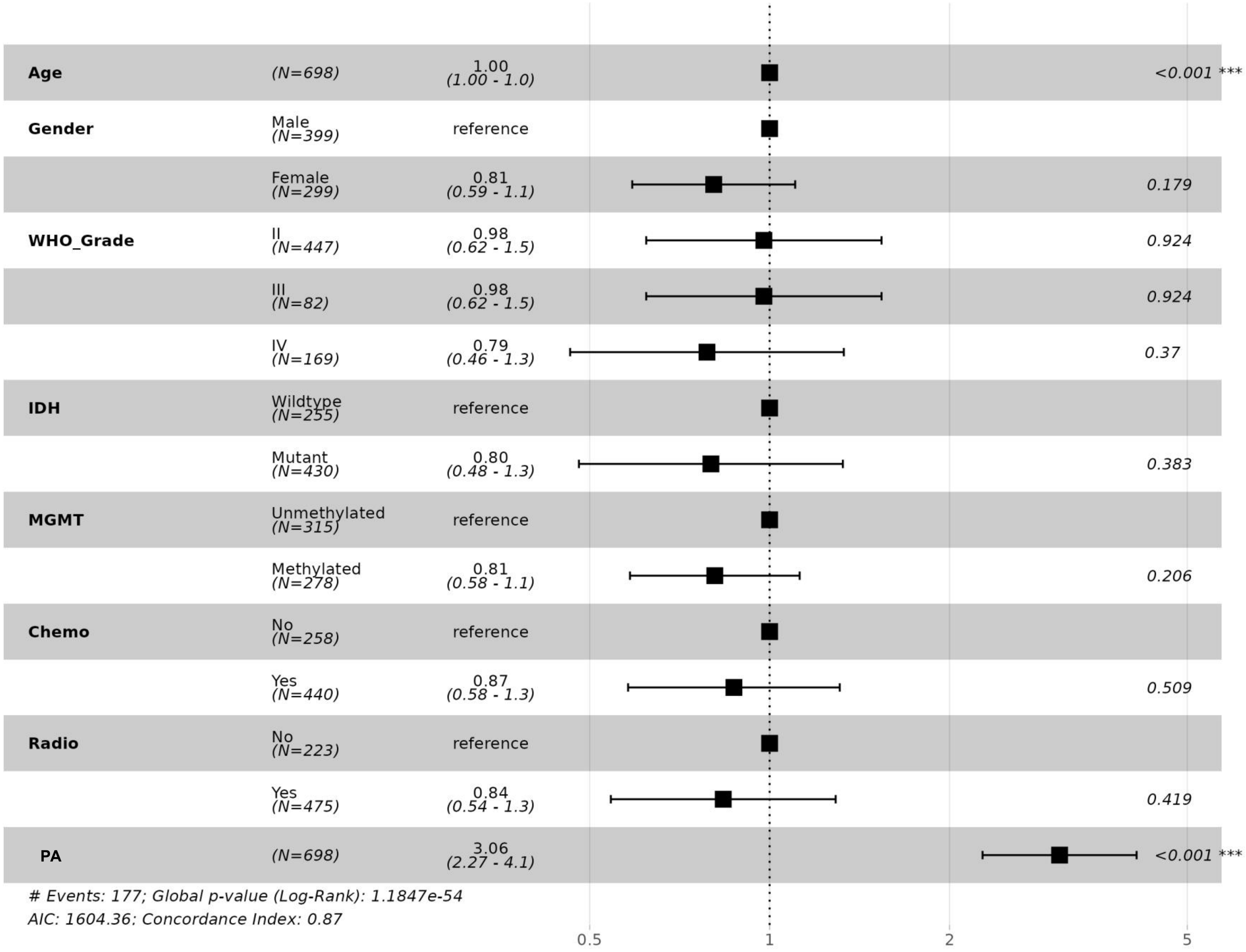

Supplement: Supplementary file 3 [file DataSheet3.pdf]
