# Supplementary material for: A cell death program–based tumor signature stratifies prognosis, immune landscape, and therapeutic response in glioma
Source: Front Oncol. 2026 May 21;16:1824504. doi: 10.3389/fonc.2026.1824504 (PMC13233278; doi:10.3389/fonc.2026.1824504)
Supplement: Supplementary file 5 [file DataSheet5.pdf]

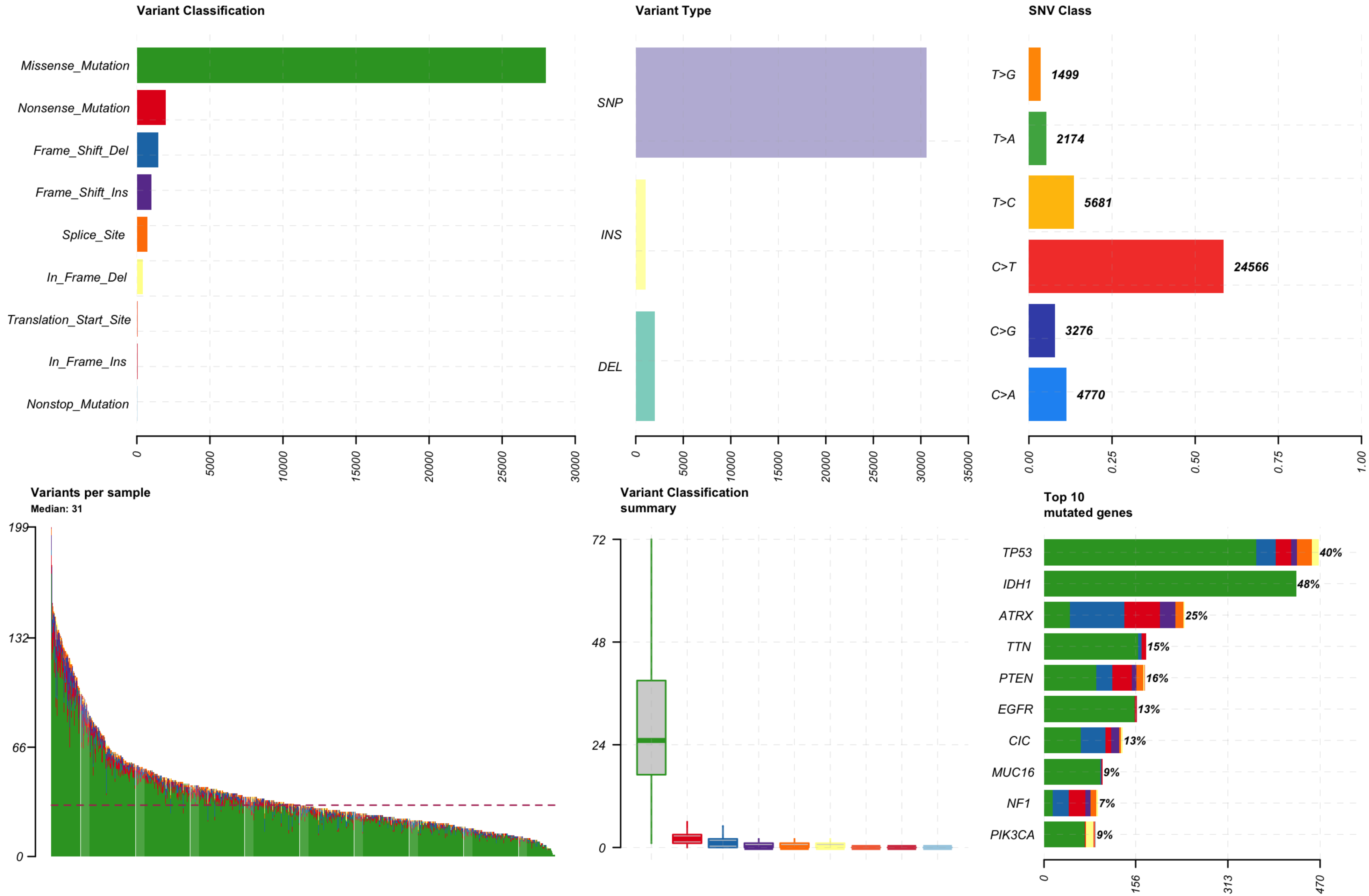

Variant Classification
Missense_Mutation
Nonsense_Mutation
Frame_Shift_Del
Frame_Shift_Ins
Splice_Site
In_Frame_Del
Translation_Start_Site
In_Frame_Ins
Nonstop_Mutation
0
5000
10000
15000
20000
25000
30000
Variant Type
SNP
INS
DEL
0
5000
10000
15000
20000
25000
30000
35000
SNV Class
T>G 1499
T>A 2174
T>C 5681
C>T 24566
C>G 3276
C>A 4770
0.00
0.25
0.50
0.75
1.00
Variants per sample
Median: 31
199
132
66
0
Variant Classification summary
72
48
24
0
Top 10 mutated genes
TP53 40%
IDH1 48%
ATRX 25%
TTN 15%
PTEN 16%
EGFR 13%
CIC 13%
MUC16 9%
NF1 7%
PIK3CA 9%
0
156
313
470
